# DELEAT: gene essentiality prediction and deletion design for bacterial genome reduction

**DOI:** 10.1186/s12859-021-04348-5

**Published:** 2021-09-18

**Authors:** Jimena Solana, Emilio Garrote-Sánchez, Rosario Gil

**Affiliations:** Institute for Integrative Systems Biology (I2SysBio), University of Valencia and CSIC, 46980 Paterna, Valencia, Spain

**Keywords:** Gene essentiality, Genome reduction, Non-model bacteria, Synthetic biology chassis

## Abstract

**Background:**

The study of gene essentiality is fundamental to understand the basic principles of life, as well as for applications in many fields. In recent decades, dozens of sets of essential genes have been determined using different experimental and bioinformatics approaches, and this information has been useful for genome reduction of model organisms. Multiple in silico strategies have been developed to predict gene essentiality, but no optimal algorithm or set of gene features has been found yet, especially for non-model organisms with incomplete functional annotation.

**Results:**

We have developed DELEAT v0.1 (*DELetion design by Essentiality Analysis Tool*), an easy-to-use bioinformatic tool which integrates an in silico gene essentiality classifier in a pipeline allowing automatic design of large-scale deletions in any bacterial genome. The essentiality classifier consists of a novel logistic regression model based on only six gene features which are not dependent on experimental data or functional annotation. As a proof of concept, we have applied this pipeline to the determination of dispensable regions in the genome of *Bartonella quintana* str. Toulouse. In this already reduced genome, 35 possible deletions have been delimited, spanning 29% of the genome.

**Conclusions:**

Built on in silico gene essentiality predictions, we have developed an analysis pipeline which assists researchers throughout multiple stages of bacterial genome reduction projects, and created a novel classifier which is simple, fast, and universally applicable to any bacterial organism with a GenBank annotation file.

**Supplementary Information:**

The online version contains supplementary material available at 10.1186/s12859-021-04348-5.

## Background

Gene essentiality is a key concept in genetics with implications ranging from basic research on the principles of life to anti-cancer therapies [1–4]. A gene is considered essential for an organism if it is indispensable for its survival, that is, if its inactivation has a lethal effect. Building on this concept, the *minimal genome* is defined as the set of genetic elements necessary and sufficient to keep alive a modern-type cellular organism in ideal conditions, i.e. in a medium containing all essential nutrients and without stresses [5]. Multiple experimental and computational methods have been used to propose a core of essential elements that must be present in a minimal genome. These attempts have followed comparative genomics strategies, manual curation of essential gene sets according to theoretically essential cellular functions, and systems approaches [6]. Moreover, systematic mutagenesis or knock-down experiments have helped determine sets of essential genes in specific organisms. These datasets have been compiled in biological databases such as the Database of Essential Genes (DEG) [7, 8]. Irrespective of the approach used and the global genome size, all essential gene sets share an approximate gene count (200–500 genes) and their contents can be mapped to three essential biological pillars [9]: the cellular genetic machinery (DNA, RNA and protein metabolism), energetic and intermediary metabolism, and cell envelope.

There exist genomes that have been naturally minimised throughout evolution due to the establishment of close symbiotic relationships. The causes and consequences of this phenomenon, known as “genome reduction syndrome”, have been studied in detail in the past decade [10]. Many of these studies, focused on bacterial endosymbionts of insects, constitute a key tool in gene essentiality research, as they prove the viability of radically simplified genomes to support cellular life and give insight into which cellular functions need to be retained even in these conditions, an indication that they must be universally essential functions [6].

Reduced genomes have also been developed in vitro through elimination of dispensable regions from natural genomes. These genome reduction projects are generally focused on organisms used as research models or of biotechnological interest, and aim either to explore the limits of genome minimisation or to optimise industrial strains for specific applications [11]. A literature review across these types of projects (Additional file 1: Table S1) shows that criteria for selection of non-essential regions in bacterial genomes are mostly based on functional annotation, comparative genomics, and essentiality classification of genes according to empirical data. Despite promising results from these projects, an increasing difficulty for further genome reduction beyond a certain point has been consistently found. This phenomenon may be attributed to unknown synthetic lethality relationships between genes [11], or under-studied negative effects of disturbing the three-dimensional chromosome structure [12]. It has been proposed that selecting a naturally reduced genome as a starting point may be advantageous, since it has already undergone a loss of genetic material in a timescale slow enough to allow evolution of robustness mechanisms that offset deleterious effects (e.g. constitutive over-expression of chaperones [13]), and are expected to show fewer synthetic lethality events [14]. On the other hand, some non-model organism may be of interest as a starting point due to intrinsic features or functionalities that are attractive for a potential application [12, 15]. In any of these cases, selection of a non-model bacterial platform for genome reduction restricts the availability of empirical data necessary for rational deletion design. Novel strategies are therefore needed for identification of dispensable genome regions.

Research lines focused on gene essentiality, minimal genomes, and large-scale genome reduction have a vast range of applications. On one hand, such studies are of crucial importance in order to define the basic fundamentals of cellular life [1, 2], a knowledge which, in turn, is valuable for the development of anti-microbial and anti-cancer therapies [3, 4]. Gene essentiality is also known to be associated with identification of human disease genes [16]. However, these studies have been most impactful in the field of synthetic biology, which holds the basic premise that comprehensive knowledge of the minimal components of a living system is the starting point for the synthesis of artificial life [3, 17]. One of synthetic biology’s main goals is to build a minimal cell which serves as a *programmable chassis*, i.e. a bare platform in which to implement any functionality of interest. Two types of strategies are proposed to achieve such chassis [18], known as bottom-up – the synthesis of a minimal cell from a set of basic known essential components – and top-down – the streamlining of a natural genome down to only its essential parts by means of large-scale deletions. Despite much relevant progress in the former [19], top-down approaches are more popular nowadays given the lesser workflow complexity and cost. A very similar concept, where large-scale genome reduction is also used as a fundamental tool, is the development of *minimum genome factories* [20] for biotechnological production of proteins or metabolites of interest. Here, sources of genomic instability (i.e. mobile genetic elements) and metabolic functions unnecessary for the specific application are deleted from the genome of industrial microorganism strains, while favourable features are retained [15, 21].

Accurate identification of essential genes is, therefore, of vital importance in many applications. However, experimental approaches are remarkably costly and slow, and may even be unfeasible for some organisms which are unculturable or when appropriate genetic manipulation techniques do not exist [22]. For these reasons, computational strategies have emerged that aim to predict whether a gene is essential given a set of known features. This is possible because the sequences of genes that are essential are differentially shaped by evolution. Most remarkably, essential genes are more highly conserved, both in the sense of there being more orthologs across diverse taxonomic groups (phyletic retention) [23] and sequence conservation (i.e. essential genes tend to evolve more slowly and be subjected to purifying selection [24], and are more likely to encode proteins with conserved domains [25]). Usually, many essential genes are also expressed at higher and less variable levels [26]. This, in turn, implies a preferential location in the leading strand of the chromosome [27] and differential values for multiple measures of codon and amino acid usage [26]. Regarding functionality, essential gene sets are enriched in functions related to the processing of genetic information [23]. In addition, essential genes tend to encode longer proteins [25, 28]. Most of the gene features mentioned so far can be deduced from the gene sequence, either directly or by comparison with databases. Some other features based on comprehensive functional annotation and experimental data, such as graph centrality measures in gene networks, have also been associated with gene essentiality [26, 29]. In summary, gene features that may be used for essentiality prediction can be classified into sequence features, sequence-derived features, and features derived from experimental data. Based on these predictive variables, many models have been developed for in silico classification of genes as essential or non-essential (Additional file 1: Table S2). Most strategies integrate multiple features in order to increase model performance, and most of them make use of supervised machine learning methods, as well. The most commonly used algorithms for this purpose are support vector machines (SVM), Naïve Bayes, decision trees and logistic regression [30]. Regarding gene features, it has been consistently found that evolutionary conservation, measured as phyletic retention, provides the greatest predictive power [30–32]. It has also been pointed out [28, 33–35] that models should strive to include only gene features that can be obtained without the need of experimental data—that is, exclusively sequence and sequence-derived features—in order to have a wider range of application, even for organisms with limited functional annotation (e.g. emergent pathogens for which it is key to find drug targets quickly).

In this article we describe DELEAT v0.1 (*DELetion design by Essentiality Analysis Tool*), an easy-to-use bioinformatic analysis pipeline that allows rational design of large-scale deletions in bacterial genomes by means of previous detection of essential genes. Essentiality prediction is performed by a novel logistic regression model based on only six gene features which are not dependent on experimental data or functional annotation. As a proof of concept, we used it to identify non-essential regions in the genome of *Bartonella quintana* strain Toulouse. This naturally-reduced 1.58 Mb genome was sequenced at the beginning of the genomics era, when the annotation tools were still not fully developed, and *ca*. 15% of its protein-coding genes lack any functional annotation.

## Implementation

### Data

All bacterial sequences and annotation data from DEG version 15.2 (essentialgene.org) were downloaded and used as reference data with known essentiality, both for the training of a gene essentiality classifier and for search of essential orthologs. These datasets were filtered to ensure their quality [36]: only the most recent experiment was selected for each species (in order to avoid bias and duplicated data in the training set), and data from plasmids and linear chromosomes, as well as from experiments in culture media other than “rich media”, were discarded. Because some datasets do not include all non-essential gene sequences, those lacking more than half of such sequences were eliminated, as well. Overall, 30 reference datasets were retained for analysis (Table 1).

**Table 1:** Reference bacterial genomes selected for this study

Organism	DEG id	RefSeq
*Acinetobacter baumannii* ATCC 17978	DEG1043	NC_009085
*Acinetobacter baylyi* ADP1	DEG1013	NC_005966
*Agrobacterium fabrum* str. C58	DEG1045	NC_003062
*Bacillus subtilis* 168	DEG1001	NC_000964
*Bacteroides fragilis* 638R	DEG1034	NC_016776
*Bacteroides thetaiotaomicron* VPI-5482	DEG1023	NC_004663
*Brevundimonas subvibrioides* ATCC 15264	DEG1046	NC_014375
*Burkholderia pseudomallei* K96243	DEG1035	NC_006350; NC_006351
*Burkholderia thailandensis* E264	DEG1024	NC_007650; NC_007651
*Campylobacter jejuni* subsp. *jejuni* 81-176	DEG1050	NC_008787
*Caulobacter crescentus*	DEG1020	NC_011916
*Escherichia coli* MG1655 II	DEG1019	NC_000913
*Francisella novicida* U112	DEG1012	NC_008601
*Haemophilus influenzae* Rd KW20	DEG1005	NC_000907
*Helicobacter pylori* 26695	DEG1008	NC_000915
*Mycobacterium tuberculosis* H37Rv III	DEG1027	NC_000962
*Mycoplasma genitalium* G37	DEG1006	NC_000908
*Mycoplasma pulmonis* UAB CTIP	DEG1014	NC_002771
*Porphyromonas gingivalis* ATCC 33277	DEG1039	NC_010729
*Pseudomonas aeruginosa* PAO1	DEG1036	NC_002516
*Rhodopseudomonas palustris* CGA009	DEG1041	NC_005296
*Salmonella enterica* serovar Typhi Ty2	DEG1033	NC_004631
*Salmonella enterica* serovar Typhimurium SL1344	DEG1032	NC_016810
*Sphingomonas wittichii* RW1	DEG1028	NC_009511
*Staphylococcus aureus* NCTC 8325	DEG1017	NC_007795
*Streptococcus agalactiae* A909	DEG1042	NC_007432
*Streptococcus pyogenes* NZ131	DEG1038	NC_011375
*Streptococcus sanguinis*	DEG1021	NC_009009
*Synechococcus elongatus* PCC 7942	DEG1040	NC_007604
*Vibrio cholerae* N16961	DEG1003	NC_002505; NC_002506

Because DEG datasets include only amino acid sequence data, and both nucleotide sequence and strand location are needed for essentiality analysis, GenBank annotation files were downloaded for all selected reference organisms. As both sources (DEG and GenBank) do not share common identifiers across their annotations, genes were mapped by sequence alignment by BLASTp search, with a cut-off of 95% sequence identity and 95% sequence length coverage.

The annotated genome of *Bartonella quintana* str. Tolouse [37] was obtained from the NCBI GenBank database (Accession number NC_005955.1).

## Pipeline design

**Fig. 1:**
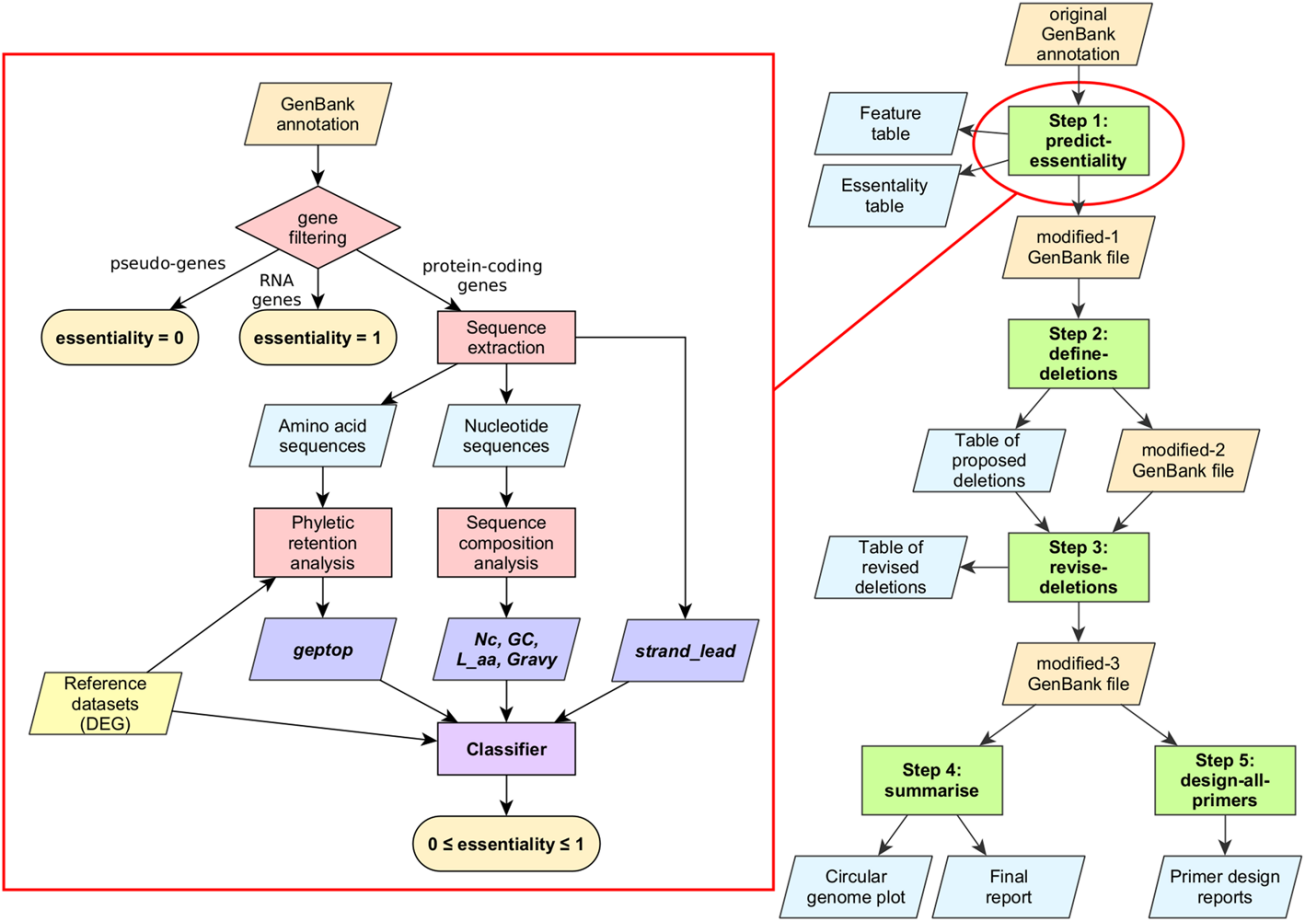
Schematic representation of the DELEAT v0.1 pipeline and gene essentiality prediction algorithm

DELEAT has been designed as a pipeline which takes a GenBank annotation file (.gb) as initial input and performs a series of analyses resulting in a list of candidate genome deletions, as well as some complementary information useful for the genome reduction process (Fig. 1). The different steps in the pipeline communicate through *modified GenBank* files which are compatible with genome visualisation tools such as Artemis [38], so that results from every step can be inspected by the user. DELEAT has been implemented for Linux systems, but can be run on any platform by means of a Docker image built from the provided Dockerfile. Software dependencies are detailed in the “Availability and requirements” section.

***Step 1: predict-essentiality*** The first step calculates an essentiality score for each annotated gene in the analysed genome (Fig. 1). This score lies in the interval [0, 1] and represents the gene’s probability of belonging to the class “essential”, which is calculated from multiple gene features (see section “Gene essentiality classifier”). These features are:

*Strand* In prokaryotes, essential genes are preferentially located in the leading strand of the chromosome, which corresponds to the “+” strand between the *ori* and *ter* coordinates (origin and terminus of replication, respectively), and to the “−“ strand on the other half (*ter* → *ori*). Therefore, these coordinates are needed in order to determine this gene feature. If they are not provided by the user, *ori* and *ter* are calculated automatically by the cumulative GC skew method [39].

*Phyletic retention (essential orthologs)* The Geptop 2.0 algorithm [32] was replicated for the calculation of this feature. For each protein in the genome, a BLASTp search is run against all reference proteomes and orthologs are identified by the reciprocal best hit (RBH) method. Once ortholog pairs are identified, a partial essentiality score is defined for protein *i* as:

$$S_{i} = \sum\limits_{j = 1}^{N} {\frac{{M_{ij} }}{{D_{j} }}}$$

where *j* is the reference proteome index, *N* is the total reference proteome count, the “mapping score” *M*_*ij*_ is 1 if an essential ortholog exists in this proteome and 0 otherwise, and *D*_*j*_ is the evolutionary distance between the problem and reference species. This distance is calculated by the composition vector method [40], based on *K*-mer frequencies in the proteome (here, *K* = 6). The composition vectors of all reference proteomes are pre-computed and provided with DELEAT in JSON format, in order to speed up the analysis. Once phyletic retention scores are obtained, they are scaled to the [0, 1] interval.

*Codon usage, G* + *C content, length and hydrophobicity score*. These gene features are calculated directly from the nucleotide sequences using the tool CodonW [41].

Scores for these 6 gene features are then pre-processed following the same steps as for the model training set, and a classifier is used to obtain essentiality scores for each gene annotated with a locus tag (see section “Gene essentiality classifier”). In the case of RNA genes and pseudogenes, they are assigned scores of 1 and 0, respectively, without the need for gene feature calculation. Essentiality scores are used for downstream analyses in the pipeline, and they are added to the genome annotation resulting in a *modified-1 GenBank* file (*.gbm1*), which can be edited to manually correct scores if necessary. Results from step 1 are also exported in CSV format.

***Step 2: define-deletions*** The second step takes a *modified-1 GenBank* file as input, together with arguments *L* (minimum desired deletion length) and *E* (score threshold for considering a gene as essential). We provide general guidelines on how to adjust these parameters in the Discussion section. A list of deletions is proposed, where a deletion is defined as a region equal to or longer than *L* which does not contain any gene with an essentiality score equal to or higher than *E*. A default non-coding margin of 200 nucleotides around essential genes is taken into account to avoid disruption of *cis*-regulatory elements, although the size of this margin may also be modified at execution. Proposed deletions are added to the genome annotation, resulting in a *modified-2 GenBank* file (*.gbm2*). A CSV file is also created with information about each deletion: name, coordinates, fraction of genome coverage, and gene content (counts of pseudogenes, hypothetical proteins, and annotated genes).

***Step 3: revise-deletions*** At this point, the user must manually curate the list of proposed deletions by accepting, rejecting, modifying or renaming each one, and/or adding new ones. Then, step 3 is run in order to update both the deletion data table and the annotation, which becomes a *modified-3 GenBank* file (*.gbm3*).

***Step 4: summarise*** Once deletions are designed, the last steps in the pipeline are dedicated to providing complementary information about the genome reduction process. This fourth step builds a circular genome map where the original genome is compared to the fully reduced one, with the use of the tool pyCircos (github.com/ponnhide/pyCircos). It also generates a report (in plain text format) which summarises the deletion design process. This report includes the selected *L* and *E* parameters, the number of genes classified based on essentiality and gene type (RNA, hypothetical, annotated, pseudogene, total), and basic data about the proposed deletions—total number of deletions, total size (in kb and % of genome) and total number of genes to be removed. Finally, an optimal deletion order is proposed according to the rule of minimising replichore imbalance at each step. A size difference large enough between the two halves of the genome delimited between *ori* and *ter* has been found to have deleterious effects on replication [1].

***Step 5: design-all-primers*** This final step is optional and serves the purpose of designing PCR primers to carry out deletions by the method of megapriming [42]. This consists in amplifying the regions flanking the desired deletion, which are then concatenated and cloned into a plasmid to be introduced into the target organism, where the cloned sequence is integrated by homologous recombination and the region in between is eliminated from the genome. Primer design for this purpose must follow a set of rules, i.e. the flanking regions should span approximately 800 bp to facilitate recombination, and the cloned product cannot contain neither any target of the restriction enzyme used for cloning nor sequences that can be found repeated in the genome. Furthermore, appropriate tail sequences must be added to the primers in order for the method to function correctly (i.e. an adequate restriction site for cloning and complementary sequences for megapriming). Given these restrictions, an algorithm was designed that automatically calculates optimal primer pairs based on the coordinates of a deletion (Additional file 1: Figure S1). It makes use of Primer3-py (libnano.github.io/primer3-py), a Python API for the primer design tool Primer3 [43]. The primer design process is detailed in an output log file which includes oligonucleotide sequences with added tails, as well as their coordinates in the genome. The flanking region sequences are also saved to a FASTA file. This step of the pipeline can be run either manually for specific deletions (*design-primers*) or automatically for all output deletions by step 3 (*design-all-primers*).

DELEAT usage documentation and code examples can be found in the GitHub repository (see “Availability and requirements” section).

### Gene essentiality classifier: training and evaluation

A logistic regression model was trained in order to classify genes according to essentiality, based on the six gene features listed in *Step 1: predict-essentiality*. The Python package scikit-learn [44] (version 0.23.1) was used for model training and fine-tuning, prediction and evaluation. We followed a supervised machine learning approach, using DEG 15.2 datasets, where genes are labelled as essential or non-essential (binary classification), as reference data. The logistic regression algorithm was chosen because it belongs to the family of linear models, which are widely used due to their interpretability and efficiency with very large datasets [45]. As such, it can be trained with the large dataset used here without the need for special computational resources, unlike other models. In addition, this algorithm is particularly well suited because it bases classification decisions on a probability value, which for this application is interpreted, and used downstream, as an “essentiality score” that allows filtering with any arbitrary threshold.

Gene feature selection was done based both on available literature and model evaluation. Among the gene features obtainable from CodonW calculations, a minimal set that optimises model performance was selected. As a measure of codon usage, we chose the effective number of codons *Nc* [46] because it does not need a reference codon usage table. From the available CodonW features we also selected GC content, protein length, and the hydrophobicity indicator *Gravy*. In addition, phyletic retention (as defined by the Geptop 2 algorithm) and strand location were determined. Once this gene feature list was defined, the complete table of all genes vs. all features was computed for each of the 30 reference organisms. The concatenation of these 30 tables was considered as our reference dataset, which was split into 60% training set and 40% test set after random shuffling.

A model training pipeline was defined as follows. First, because the feature *Nc* sometimes cannot be calculated for short genes, missing data are imputed – with the default method, the average of observed values. Then, all data are scaled to the [0, 1] interval, given that the regularised logistic regression model requires all variables to be in the same range. Finally, the model is trained on the training dataset, using L2 regularisation to avoid overfitting, and rebalancing due to class imbalance (there are many more non-essential genes than essential). After designing this training protocol, a grid search was carried out to find the optimal value of *C*, the regularisation strength parameter.

Model evaluation was based on the area under the ROC curve (AUC) statistic, which is a well-suited metric for datasets with class imbalance and is a standard in the gene essentiality classification literature. Evaluation was carried out with three different approaches: calculating the AUC only for the test set, with fivefold cross-validation, and with leave-one-species-out cross-validation.

## Results

### Gene essentiality classifier

*Calculation of reference dataset and model training* Computing of the six selected features for all genes labelled with a DEG identifier in the 30 selected reference organisms resulted in 91,748 total data points which were used for model training and evaluation, of which 79,906 are essential genes and 11,842 are non-essential. Value distributions of the six features for all reference genes are shown in Additional file 1: Figure S2.

A logistic regression classifier with L2 regularisation was trained on 60% of the reference dataset. This is a simple model, with only six features which can be deduced from gene sequences and which have low correlation among each other (Additional file 1: Table S3). Model coefficients (Additional file 1: Table S4) suggest that the phyletic retention feature bears most of the weight in classification decisions, which is in line with results obtained by Dong and co-workers [30] after assessing the integration of multiple indicators for gene essentiality prediction in prokaryote genomes.

**Fig. 2:**
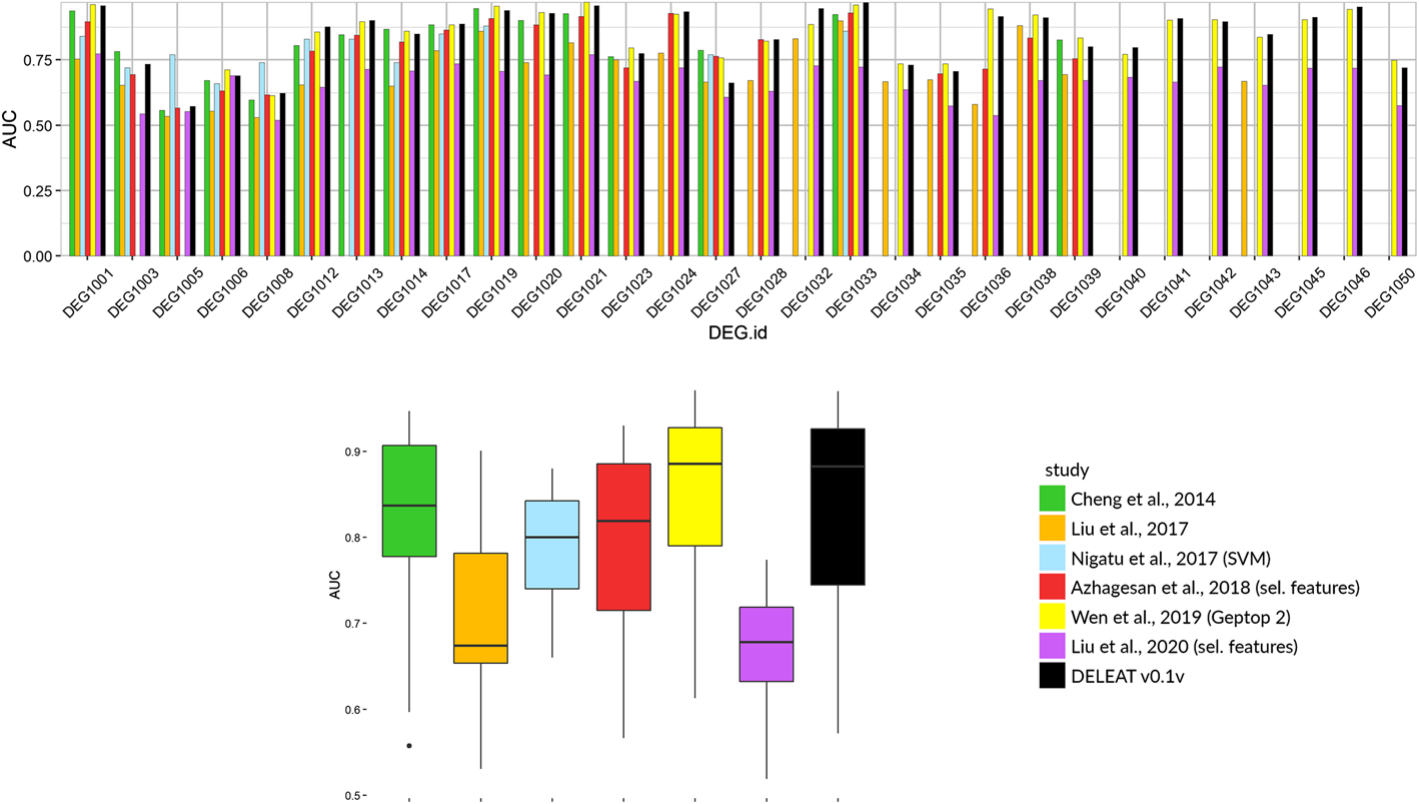
AUC scores obtained from leave-one-species-out model cross-validation. We compared our model with other 6 different strategies from the literature, which take into account evolutionary conservation only (Geptop 2); 40 (Liu et al. 2017, [40]), 91 (Nigatu et al. 2017, [41]) or 90 sequence features (Liu et al. 2020, [54]); 100 sequence and topology features (Azhagesan et al. 2018, [53]), or 15 sequence, topology and gene expression features (Cheng et al. 2014, [42]). AUC values are plotted organism-wise in the upper panel to illustrate the differences among models for each reference species, and aggregated for each model in the lower panel to show overall score distributions

*Model evaluation* On one hand, an AUC score of 0.8401 was obtained for the model when evaluated on the test set (Additional file 1: Figure S3), and fivefold cross-validation yielded AUC values of 0.8458 ± 0.005. In addition, we tested predictions following a leave-one-species-out protocol, where the model is iteratively trained on the data from all reference species except one, and tested on the left-out species. AUC values resulting from this evaluation (Additional file 1: Table S5) are shown in Fig. 2, compared with other prediction models from the literature [32, 34–36, 47, 48]. Prediction scores for DELEAT’s classifier are generally comparable to the other tools, and the best so far for 11 of the 30 reference organisms (Additional file 1: Table S5). Yet, the average AUC value for Geptop 2 is slightly higher than our classifier’s (0.838 ± 0.098 vs 0.834 ± 0.112), and the former performs the best for 13/30 organisms. We further compare results from both tools in the following section (“Proof of concept”) and argue that DELEAT shows a better general applicability (see “Discussion”). Remarkably, some of the classifiers included in the comparison and which obtained similar prediction scores are much more complex, include features obtained from experimental data, and are presumably more computationally intensive. This evidences the potential of using exclusively sequence-derived features and a streamlined prediction model to obtain good-quality gene essentiality predictions.

### Proof of concept: genome analysis of *Bartonella quintana* str. Toulouse

*B. quintana* str. Toulouse, the causative agent of trench fever, was used as a model for the validation test of our tool. *Bartonella* spp. are vector-borne bacteria that infect mammalian erythrocytes. This genus was proposed a decade ago as a suitable model to design a customized endosymbiont chassis that could be used as a drug delivery system [54]. To do so, the genome needs to be stripped of all unnecessary genes, as well as those involved in virulence against the selected host.

The organism’s GenBank annotation file was used for analysis with DELEAT. Besides this, the only additional information needed as input are the genome replication origin (*ori*) and termination (*ter*) coordinates (1,581,000 and 723,000, respectively) and the name of the restriction enzyme to be used for vector cloning (BamHI in this case). Multiple essentiality score thresholds were explored taking into account the size of the resulting essential gene set. A cut-off of 0.75 was finally selected as it classified 460 genes as essential, a number comparable to gene sets obtained by experimental methods for other organisms. A minimum deletion length needs to be chosen based on the length of the genome, as small deletions will not have relevant consequences on the replichores equilibrium. We chose a minimum of 7000 bp, which is about 0,5% of the genome size of our model *B. quintana* (1.5 Mb). All analyses were run on a laptop with Ubuntu 18.04.3 as the operating system, 4 GB RAM and Intel® Core™ i7-4510U 2.00 GHz CPU (2 cores with hyper-threading).

With an essentiality score threshold of 0.75, 460 genes were identified as essential in the first step of the pipeline, of which 408 are protein-coding and 52 are RNA genes. This set of genes was compared to the Core Minimal Genome (CMG) as defined by Gil et al. [6, 9] as a means of validation (Additional file 1: Table S6), since no experimental data exists on gene essentiality for this organism. Genes involved in metabolism and membrane transport were excluded from the comparison, because transporters were not taken into account in the CMG and it is already known that there are many alternative minimal metabolisms [6]. Of 158 genes that were checked, 139 were identified as essential in *B. quintana*’s genome by our classifier (i.e. 88% sensitivity). Five of the 19 genes that DELEAT classified as non-essential were, in fact, described as putatively non-essential in the CMG. Additionally, we selected 59 *B. quintana* genes that we had previously classified as involved in pathogenicity or prophage-related, to use them as a reference set of non-essential genes. Among them, 56 were correctly classified by DELEAT. The 19 misclassified essential genes (false negatives) and the three pathogenicity-related genes misclassified as essential (false positives) were modified accordingly in the annotation for the subsequent pipeline steps, by manually correcting the essentiality scores in the modified-1 GenBank file (*.gbm1*).

In addition, gene essentiality prediction results were compared with those obtained by Geptop 2 for the same organism, using the default score threshold of 0.24 and comparing only protein-coding genes (the only ones analysed by Geptop 2). Of 408 protein-coding genes identified as essential by DELEAT, 336 (82%) are labelled equally by Geptop 2. Approximately half of the 44 genes classified as essential by Geptop 2 but not by DELEAT are involved in metabolic pathways, most of which are incomplete in the reduced metabolism of *B. quintana*, based on the data obtained from KEGG database [50, 51] and, therefore, non-essential as their products can be obtained from the mammalian host. Essentiality prediction through ortholog mapping labels these genes as essential because, in most free-living species, these pathways are needed for survival. Therefore, only a multiple-feature strategy can identify this type of non-essential genes. On the other hand, genes classified as essential only by DELEAT are enriched in “hypothetical protein” and “domain of unknown function” annotations. Through BLASTp search, we found one third of these to have conserved orthologs in other species, mostly restricted to the order Rhizobiales. This suggests that they may be taxon-specific essential genes, which cannot be identified as such by ortholog mapping if there are no orthologs among the selected reference species, but exhibit other gene features which reveal them as essential. In short, integration of evolutionary conservation (by ortholog mapping with BLASTp) with other five gene features (chromosome strand location, codon usage, GC content, protein length and hydrophobicity) improves predictive power, as has been pointed out before [30], particularly for species-specific essential and non-essential genes.

Execution of the *define-deletions* step with parameters *E* = 0.75 and *L* = 7000 yielded a list of 41 proposed deletions (Additional file 1: Table S7), spanning a total length of 509.9 kb (32.3% of the genome) and averaging 12.4 ± 5.6 kb. Several of them match deletions that were previously defined by manual inspection of functional annotation data in search of putative horizontally acquired regions. In fact, three of the proposed deletions correspond to the three pathogenicity islands which have been described in *B. quintana*’s genome (Additional file 1: Figure S4) – the Vomp (variably expressed outer membrane proteins) locus, a type V secretion system comprising multiple adhesins; the type IV secretion system VirB/VirD4 locus including the adjacent Bep effectors (*Bartonella* effector proteins), which are involved in endothelial cell invasion, and the type IV secretion system Trw locus, responsible for intra-erythrocytic parasitism [52].

To test operation of step 3, we performed a manual curation of the set of proposed deletions and selected seven to be removed from the list, as they contained a DNA recombination system – which is necessary for a genome reduction process – and several membrane transporters. This left a total of 35 deletions with an average length of 12.9 ± 5.9 kb and a total of 452.8 kb (28.6% of the genome).

**Fig. 3:**
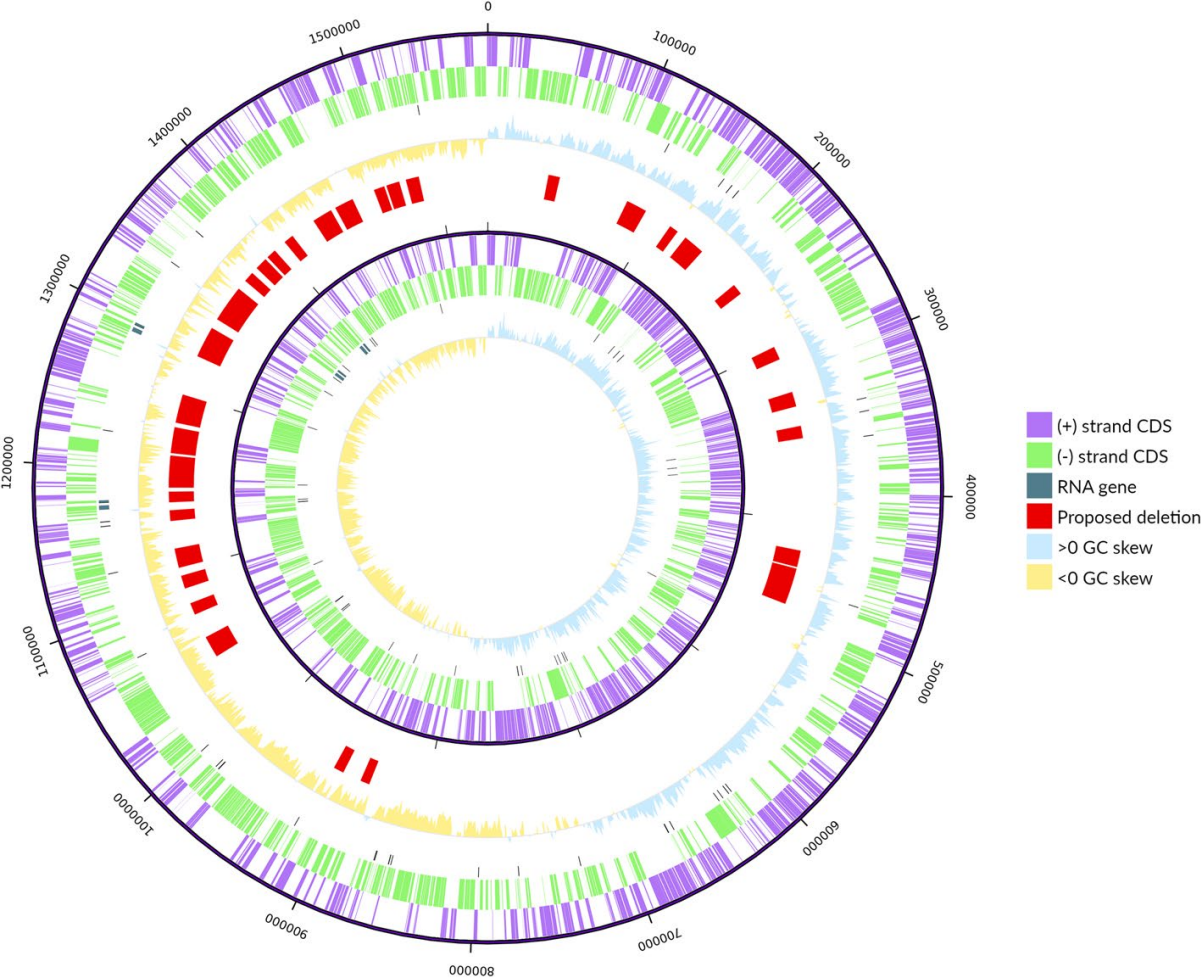
Comparison of original (exterior rings) and reduced (interior rings) genomes, according to proposed deletions. In each case, protein-coding genes in the + and – strands are shown in purple and green, respectively; RNA genes are represented in black, and a GC skew graph (100,000 bp window and 100,000 bp step) is included, as well. In between both genomes, proposed deletions are shown in red

Execution of the *summarise* step produced a circular genome map displaying the potential genome reduction, which is shown in Fig. 3. According to the automatically generated report, the essential gene count of the complete genome is 476, of which 52 are RNA genes and 34 have no functional annotation. The non-essential gene count is 912, of which 115 are pseudogenes and 162 encode “hypothetical proteins”. 492 of these non-essential genes are found within the 35 designed deletions. In addition, the report indicates an ideal order for deletion execution that minimises replichore size imbalance at each step.

Finally, the *design-all-primers* command was used for automatic PCR primer design. Repetitive regions and restriction enzyme target sites were successfully detected by the algorithm, and a set of appropriate PCR primers was generated in approximately one second for each deletion.

## Discussion

DELEAT v0.1 aims to be a useful tool for bacterial genomics, particularly in the early stages of genome reduction projects for any application. The pipeline includes a novel gene essentiality prediction model, in the form of a logistic regression classifier trained on datasets from DEG 15.2 and with only six sequence-derived gene features.

Depending on genome size, the complete DELEAT pipeline can be executed in approximately an hour on a standard personal computer running Linux. For organisms with a small genome such as *B. quintana* str. Toulouse (1.58 Mb), it can take as little as 15 min. The first step in the pipeline (*predict-essentiality*) comprises most of the total calculations, and therefore takes the longest to run (the execution time was 14:05 min in this example, running the essential orthologs search with 4 threads). Our novel implementation of the Geptop 2 algorithm improves efficiency relative to the original one. On one hand, the inclusion of pre-calculated composition vectors for all reference proteomes notably speeds up the analysis, since only the reference species composition vector needs to be computed at runtime. It also makes use of the native BLAST + job concurrency in threads instead of parallelising Python code, which has a lower computational cost. All things considered, the new implementation can be run faster and with less memory usage.

As opposed to other gene essentiality prediction tools, step 1 of DELEAT provides scores for all genes annotated in a GenBank file, including non-coding genes. This allows the determination of non-essential regions in the genome directly based on results from this step. Regarding deletion design parameters, we recommend exploring different values of *E* by comparing the resulting essential gene set size to sets determined experimentally in the literature, taking into account that raising the cut-off value will minimise false positives and lowering it will reduce false negatives, the latter being a more conservative approach from the perspective of genome reduction (i.e. lesser probability of eliminating a gene that is essential). The *L* parameter can be explored by visually inspecting the proposed deletions on the genome of interest. We suggest taking into account the relative size of the complete genome, as large deletions can cause replichore imbalances during replication and have deleterious effects.

It is worth noting that the precise coordinates of each deletion in the genome are ultimately dependent on primer design, and the values given by DELEAT at the *propose-deletions* step correspond to the longest possible span. These coordinates are the ones used to generate the circular genome map in step 4.

The gene essentiality classification model developed here heavily relies on evolutionary conservation as a predictive gene feature, which is known to be the best indicator in prokaryotic genomes [30]. However, it also integrates other sequence-derived features with the aim of overcoming the limitations of exclusively using phyletic retention – and, in general, of inferring gene essentiality from comparative genomics strategies. Widespread conservation of a gene does not necessarily imply that it is essential [53], since non-orthologs can fulfil the same essential function in different organisms (i.e. non-orthologous gene displacement), and very divergent orthologs may not be identified as such because of low sequence identity. Most remarkably, the predictive power of phyletic retention decays with evolutionary distance between the query and the reference species, and homology mapping is inherently unfit for the identification of species-specific essential genes [25, 29–31]. Several other essentiality prediction models have previously been developed that also integrate evolutionary conservation with other gene features [25, 28, 33, 42, 54–57]. Analysis of gene essentiality predictions in *B. quintana* str. Toulouse suggests that our classifier succeeds in improving these limitations, while being based on a very simple model with easy-to-calculate gene features, both in the sense of requiring little annotation data and of computational speed.

AUC scores from leave-one-species-out cross-validation for DELEAT’s classifier are very similar to those for Geptop 2, which is explained by the fact that this feature is included in DELEAT’s logistic regression model and has a strong weight in classification. However, by integrating other gene features, an essentiality score is provided for every analysed gene, unlike the Geptop 2 algorithm, which assigns a score of 0 to all genes lacking any essential ortholog among the reference organisms. Because this is the case for more than half of the genes studied here, it can be argued that DELEAT’s classifier is globally more informative. Furthermore, it has the potential to avoid false negatives among essential genes which do not have essential orthologs in the reference species but can be identified as such by analysis of other gene features, and species-specific essential genes in general. This model design may also prevent false positives, as a gene which is largely evolutionarily conserved may not be essential in a particular organism, which can be detected through the rest of gene features included here (e.g. low GC content, location in the lagging strand, etc.).

Because the essentiality analysis performed by DELEAT is based on sequence-derived features alone, this tool is applicable to any bacterial species having a GenBank annotation file, even if functional annotation of the genome is not comprehensive. Nonetheless, manual curation of results is strongly encouraged, as in silico gene essentiality classification tools, no matter how accurate the algorithm, are bound to yield some false positives and, remarkably, false negatives, which must be avoided at all costs in a genome reduction project. As a limitation, the essentiality analysis performed by DELEAT is done on a gene-by-gene basis, an approach which is unable to take into account gene interactions that may lead to synthetic lethality events.

Finally, it is worth noting that the later sections of the pipeline can be useful beyond the canonical use of DELEAT, e.g. starting at step 2 (*define-deletions*) from a GenBank file containing essentiality scores obtained with a different tool, using only step 4 (*summarise*) to build a circular genome map from a list of deletions determined by any other means, or running step 5 (*design-primers*) to perform PCR primer design for deletion of any desired genome region.

## Conclusions

Building on the idea of in silico gene essentiality prediction, we have developed a tool that makes use of these predictions to define contiguous regions in a bacterial genome which can be considered non-essential and, therefore, are deletion candidates in the context of genome minimisation efforts. Our tool allows classification of all genes in a bacterial genome according to essentiality, automatic design of large-scale genome deletions based on this data, and assistance in the genome reduction process through complementary information. DELEAT incorporates a novel logistic regression model for in silico gene essentiality prediction, which is based on six sequence-derived gene features and provides AUC values competitive with other, more complex models in the literature. Finally, we have applied our tool to the analysis of the genome of a non-model bacterial organism, *Bartonella quintana* str. Toulouse, and shown its potential for rational, automatic design of genome deletions in the context of lack of full functional annotation. We hope DELEAT will be valuable for researchers in the field of bacterial genomics, and encourage its use even if only as a complementary source of information, given its ease of installation and use.

### Availability and requirements

- **Project name:** DELEAT.
- **Project home page:**https://github.com/jime-sg/deleat.
- **Operating system(s):** Linux.
- **Programming language:** Python.
- **Other requirements:** Python (python.org) 3.7.6, BLAST + (blast.ncbi.nlm.nih.gov/Blast.cgi?PAGE_TYPE=BlastDocs&DOC_TYPE=Download)2.9.0, CodonW (codonw.sourceforge.net) 1.4.4, Vmatch (vmatch.de) 2.30, Artemis (sanger.ac.uk/tool/artemis) 18.1.0, Biopython (biopython.org) 1.77, Joblib (joblib.readthedocs.io) 0.15.1, Matplotlib (matplotlib.org) 3.2.2, more-itertools (more-itertools.readthedocs.io) 8.4.0, NumPy (numpy.org) 1.18.5, pandas (pandas.pydata.org) 1.0.5, Primer3-py (libnano.github.io/primer3-py) 0.6.0, PyCircos (github.com/ponnhide/pyCircos), and scikit-learn (scikit-learn.org) 0.23.1. All dependencies are available as packages through the management system Conda (docs.conda.io) and can be installed automatically in a Conda environment as detailed in the documentation. A Dockerfile is also provided which allows to build a Docker image with all dependencies, allowing for cross-platform execution. License: GNU GPL. Any restrictions to use by non-academics: none.

## Supplementary Information

**Additional file 1:Table S1**. Overview of bacterial genome reduction projects in the literature. **Table S2**. Gene features used for in silico essentiality prediction in the literature. **Table S3**. Correlation between model variables (Pearson coefficient). **Table S4**. Model coefficients (signs correspond to prediction of the class “non-essential”). **Table S5**. Comparison of leave-one-species-out AUC values with other models from the literature. Highlighted in yellow, the reference organisms for which DELEAT gives the best prediction scores. **Table S6**. Genes from *B. quintana*’s genome classified as essential by DELEAT. Their presence in the Core Minimal Genome proposed by Gil et al. (2004) is indicated. **Figure S1**. Algorithm for PCR primer design in the DELEAT pipeline. Starting from a proposed deletion in the genome, a pair of 1000 bp-long “margins” is defined around the start and end coordinates, with 200 bp inside of the deletion and 800 outside. Next, two conditions are checked—that the margins do not contain neither target sites of the restriction enzyme selected for cloning, nor sequences longer than 20 bp that are repeated in the genome (in order to avoid undesired homologous recombination events). If any of these conditions is violated, the affected margin is shifted “inwards” (right in the case of margin 1 and left for 2) until the problematic position falls outside it. This process is repeated until both margins comply with the primer design rules. Once the final margin coordinates are obtained, functions from the Primer3-py package are used to generate a list of the best 20 primer pairs inside the margins. The best pair of PCR products is then decided as those generated from the best primer pairs and which do not form a restriction target site when concatenated, or have a size difference larger than 400 bp. These are the PCR products that will be used for the megapriming reaction. **Figure S2**. Value distributions of the six computed features for all genes in the training and test sets. **Figure S3**. ROC curve obtained by model evaluation on the test set. **Figure S4**. Designed deletions (in green) which match pathogenicity islands in *B. quintana*’s genome, visualised in Artemis. D6 (176687 – 193839) matches the Vomp locus, D27 (1249540–1272464) the VirB/VirD4 locus, and D37 (1463545 – 1478903) the Trw locus.

## Data Availability

DELEAT is available at https://github.com/jime-sg/deleat.

## Abbreviations

AUC: Area under the ROC curve
CMG: Core minimal genome
CSV: Comma-separated values
DEG: Database of essential genes
DNA: Deoxyribonucleic acid
DELEAT: DELetion design by Essentiality Analysis Tool
JSON: JavaScript object notation
PCR: Polymerase chain reaction
RNA: Ribonucleic acid
