# Supplementary material for: DELEAT: gene essentiality prediction and deletion design for bacterial genome reduction
Source: BMC Bioinformatics. 2021 Sep 18;22:444. doi: 10.1186/s12859-021-04348-5 (PMC8449488; doi:10.1186/s12859-021-04348-5)
Supplement: Supplementary file 1 — Additional file 1:Table S1. Overview of bacterial genome reduction projects in the literature. Table S2. Gene features used for in silico essentiality prediction in the literature. Table S3. Correlation between model variables (Pearson coefficient). Table S4. Model coefficients (signs correspond to prediction of the class “non-essential”). Table S5. Comparison of leave-one-species-out AUC values with other models from the literature. Highlighted in yellow, the reference organisms for which DELEAT gives the best prediction scores. Table S6. Genes from B. quintana’s genome classified as essential by DELEAT. Their presence in the Core Minimal Genome proposed by Gil et al. (2004) is indicated. Figure S1. Algorithm for PCR primer design in the DELEAT pipeline. Starting from a proposed deletion in the genome, a pair of 1000 bp-long “margins” is defined around the start and end coordinates, with 200 bp inside of the deletion and 800 outside. Next, two conditions are checked—that the margins do not contain neither target sites of the restriction enzyme selected for cloning, nor sequences longer than 20 bp that are repeated in the genome (in order to avoid undesired homologous recombination events). If any of these conditions is violated, the affected margin is shifted “inwards” (right in the case of margin 1 and left for 2) until the problematic position falls outside it. This process is repeated until both margins comply with the primer design rules. Once the final margin coordinates are obtained, functions from the Primer3-py package are used to generate a list of the best 20 primer pairs inside the margins. The best pair of PCR products is then decided as those generated from the best primer pairs and which do not form a restriction target site when concatenated, or have a size difference larger than 400 bp. These are the PCR products that will be used for the megapriming reaction. Figure S2. Value distributions of the six computed features for all genes in the training [file 12859_2021_4348_MOESM1_ESM.doc]

**ADDITIONAL FILE 1**

**DELEAT: gene essentiality prediction and deletion design for bacterial genome reduction**

Jimena Solana^1^, Emilio Garrote-Sánchez^1^ and Rosario Gil^1^,*

^1^ Institute for Integrative Systems Biology (I2SysBio), University of Valencia and CSIC, 46980 Paterna (Valencia), Spain.

**SUPPLEMENTARY TABLES**

**Supplementary Table S1**. Overview of bacterial genome reduction projects in the literature.

| **Organism** | **Reduction** | **Criteria for deletion design** | **Phenotype** | **Reference** |
| --- | --- | --- | --- | --- |
| *Escherichia coli* | 12 deletions; 409 genes; 8%; 376 kb | Island identification by comparison to other strains | Growth and competence unchanged | Kolisnychenko *et al*., 2002 |
| *Escherichia coli* | 16 deletions; 30%; 1.4 Mb | Essentiality annotation from experimental data (PEC database) | Slower growth, altered cell morphology and chromosome organisation | Hashimoto *et al*., 2005 |
| *Escherichia coli* | 43 deletions; 743 genes; 15%; 708 kb | Functional annotation: large genomic islands and mobile genetic elements | Growth comparable to initial, better electroporation efficiency, better genome stability, heterologous protein production unchanged | Pósfai *et al*., 2006 |
| *Escherichia coli* | 53 deletions; 1081 genes; 22%; 1 Mb | Selection of non-essential genes by comparison with *Buchnera* sp. APS and from experimental data (PEC and ERGO databases). Candidate regions: >10 consecutive non-essential genes. Additionally, mobile genetic elements and toxin-antitoxin pairs. | Better growth, greater production of threonine | Mizoguchi *et al*., 2008 |
| *Escherichia coli* | 33 deletions; 39%; 1.8 Mb | Not specified | More sensitive to oxidative stress | Iwadate *et al*., 2011 |
| *Escherichia coli* | 63/92 deletions; 28/35%; 1.3/1.6 Mb | Functional annotation, comparative study with *Shigella* strains, unknown function genes, deletions from other studies. | Better growth and genome stability (completely IS-free) | Hirokawa *et al*., 2013 |
| *Escherichia coli* | 56 deletions; 23%; 1.1 Mb | Functional annotation: mobile genetic elements and islands; flagella, fimbriae and lipopolysaccharide synthesis genes | Growth comparable to initial, better genome stability (completely IS-free) | Park *et al*., 2014 |
| *Bacillus subtilis* | 6 deletions; 332 genes; 8%; 320 kb | Functional annotation, G+C content, codon bias and ORF length (>85 aa). Deletion of two prophages, three prophage-like elements, and an antibiotic biosynthesis operon. | Healthy and viable cells, physiology practically unchanged | Westers *et al*., 2003 |
| *Bacillus subtilis* | 17 deletions; 24%; 991 kb | Functional annotation (prophages, prophage-like elements, secondary metabolite biosynthesis genes) and experimental essentiality data | Unstable growth, morphology and protein production | Ara *et al*., 2007 |
| *Bacillus subtilis* | 11 deletions; 865 genes; 21%; 874 kb | Functional annotation. Deletion of prophages, prophage-like elements and secondary metabolite biosynthesis genes. | Slower growth, better heterologous protein production | Morimoto *et al*., 2008 |
| *Bacillus subtilis* | 18/21 deletions; 18/19%; 756/814 kb | Functional annotation: prophages, antibiotic biosynthesis operons and other "miscellaneous" non-essential regions | Better growth, lower competence, greater production of timidine and guanosine | Li *et al*., 2016 |
| *Bacillus subtilis* | 94/88 deletions; 1605/ 1553 genes; 36/35%; 1.5/1.4 Mb | Previous comprehensive study defining a minimal genome (Reuß *et al*., 2016). Essentiality annotation from literature, evolutionary conservation and expression level. | Viable cells with growth rates comparable to initial | Reuß *et al*., 2017 |
| *Corynebacterium glutamicum* | 3 deletions; 73 genes; 2%; 67 kb | Identification of >10 kb islands (by comparison with another strain) | Unchanged | Suzuki *et al*., 2005a |
| *Corynebacterium glutamicum* | 8 deletions; 188 genes; 6%; 190 kb | Identification of >10 kb islands (by comparison with another strain) | Unchanged | Suzuki *et al*., 2005b |
| *Corynebacterium glutamicum* | 3 deletions; 6%; 197 kb | Functional annotation: deletion of all three prophages | Better growth in stress conditions, competence and protein production | Baumgart *et al*., 2013 |
| *Corynebacterium glutamicum* | 13 deletions; 412 genes; 13%; 440 kb | Previous comprehensive study (Unthan *et al*., 2015). Essentiality annotation from literature, evolutionary conservation and expression level. | Growth unchanged | Baumgart *et al*., 2018 |
| *Streptomyces avermitilis* | 1 deleción; 17-18%; 1.4-1.5 Mb | Sub-telomeric region much bigger than close species, containing mobile genetic elements and secondary metabolite biosynthesis genes. | Better genome stability, greater production of multiple secondary metabolites | Komatsu *et al*., 2010 |
| *Pseudomonas putida* | 11 deletions; 300 genes; 4%; 262 kb | Functional annotation: mobile genetic elements, flagellar machinery and defence systems against foreign genetic material | Better growth, viability, plasmid stability and heterologous protein production (Lieder *et al*., 2015) | Martínez-García *et al*., 2014 |
| *Lactococcus lactis* | 4 deletions; 114 genes; 3%; 72 kb | Functional annotation: four regions containing prophage sequences and of unknown function | Better growth, greater heterologous protein production | Zhu *et al*., 2017 |
| *Bacillus amyloliquefaciens* | 7 deletions; 133 genes; 4%; 168 kb | Functional annotation and G+C content. Genomic islands, prophage sequences, non-essential biosynthetic pathways | Better growth, transformation efficiency, heterologous protein production, and production of surfactin | Zhang *et al*., 2020 |
| *Pseudomonas mendocina* | 14 deletions; 8%; 418 kb | Comparison with DEG database | Growth comparable to initial, better transformation efficiency, greater production of polyhydroxyalkanoate and alginate oligosaccharide | Fan *et al*., 2020 |
| *Magnetospirillum gryphiswaldense* | 16 deletions; 6%; 228 kb | Functional annotation; identification of mobile genetic elements by homology search, and of prophages by PHAST tool | Growth and transformation efficiency comparable to initial, better genome stability | Zwiener *et al*., 2021 |

**Supplementary Table S2**. Gene features used for *in silico* essentiality prediction in the literature.

| **Sequence features** | | |  | **Sequence-derived features** | | | | |  | **Features derived from experimental data** | | | | |  |
| --- | --- | --- | --- | --- | --- | --- | --- | --- | --- | --- | --- | --- | --- | --- | --- |
| **Feature** | **References** | |  | **Feature** | | **References** | | |  | **Feature** | | | **References** | |  |
| length | Gustafson *et al*., 2006; Hwang *et al*., 2009; Deng *et al*., 2011; Cheng *et al*., 2014; Lin *et al*., 2017; Liu *et al*., 2017; Liu *et al*., 2020 | |  | phyletic retention | | Gustafson *et al*., 2006; Hwang *et al*., 2009; Holman *et al*., 2009; Plaimas *et al*., 2010; Deng *et al*., 2011; Lin y Zhang, 2011; Wei *et al*., 2013; Cheng *et al*., 2014; Song *et al*., 2014; Lin *et al*., 2017 | | |  | topological properties | | | da Silva *et al*., 2008; Hwang *et al*., 2009; Plaimas *et al*., 2010; Deng *et al*., 2011; Cheng *et al*., 2014; Lin *et al*., 2017 | |  |
| strand | Gustafson *et al*., 2006; Hwang *et al*., 2009; Lin y Zhang, 2011; Song *et al*., 2014; Liu *et al*., 2017 | |  | functional domains | | Deng *et al*., 2011; Cheng *et al*., 2014; Song *et al*., 2014; Lin *et al*., 2017; Liu *et al*., 2020 | | |  | gene expression | | | Plaimas *et al*., 2011; Deng *et al*., 2011; Cheng *et al*., 2014 | |  |
| G+C content | Ning *et al*., 2014; Lin *et al*., 2017; Liu *et al*., 2017; Liu *et al*., 2020 | |  | paralogs | | Gustafson *et al*., 2006; Deng *et al*., 2011; Cheng *et al*., 2014; Song *et al*., 2014 | | |  |  | | |  | |  |
| codon usage | Gustafson *et al*., 2006; Plaimas *et al*., 2011; Deng *et al*., 2011; Lin y Zhang, 2011; Cheng *et al*., 2014; Ning *et al*., 2014; Song *et al*., 2014; Lin *et al*., 2017; Liu *et al*., 2017; Liu *et al*., 2020 | |  |  | | |  | |  |  | | |  | |  |
| amino acid usage | Plaimas *et al*., 2011; Deng *et al*., 2011; Ning *et al*., 2014; Song *et al*., 2014; Liu *et al*., 2017; Liu *et al*., 2020 |  | | |  | | |  | | |  |  | |  | |
| location (predicted) | Deng *et al*., 2011; Liu *et al*., 2017; Liu *et al*., 2020 |  | | |  | | |  | | |  |  | |  | |
| other | Li *et al*., 2017; Nigatu *et al*., 2017; Liu *et al*., 2020 |  | | |  | | |  | | |  |  | |  | |

**Supplementary Table S3**. Correlation between model variables (Pearson coefficient).

|  | **strand_lead** | **geptop** | **Nc** | **GC** | **L_aa** | **Gravy** |
| --- | --- | --- | --- | --- | --- | --- |
| **strand_lead** | 1 |  |  |  |  |  |
| **geptop** | 0.0504 | 1 |  |  |  |  |
| **Nc** | 0.0422 | -0.0865 | 1 |  |  |  |
| **GC** | -0.0646 | -0.0783 | -0.5655 | 1 |  |  |
| **L_aa** | 0.0099 | 0.0441 | -0.0507 | 0.0584 | 1 |  |
| **Gravy** | -0.0135 | -0.0808 | -0.0841 | 0.1427 | -0.0212 | 1 |

**Supplementary Table S4**. Model coefficients (signs correspond to prediction of the class “non-essential”).

| **Variable** | **Coefficient** |
| --- | --- |
| (intercept) | -0.2936 |
| *strand_lead* | 0.0885 |
| *geptop* | -7.2551 |
| *Nc* | 0.7833 |
| *GC* | 1.6506 |
| *L_aa* | 0.0242 |
| *Gravy* | -0.1122 |

**Supplementary Table S5**. Comparison of leave-one-species-out AUC values with other models from the literature. Highlighted in yellow, the reference organisms for which DELEAT gives the best prediction scores.

| **DEG id** | **Organism** | **Cheng *et al*., 2014** | **Liu *et al*., 2017** | **Nigatu *et al*., 2017**  **(SVM)** | **Azhagesan *et al*., 2018 (selected features)** | **Wen *et al*., 2019**  **(Geptop 2)** | **Liu *et al*., 2020 (selected features)** | **DELEAT v0.1** |
| --- | --- | --- | --- | --- | --- | --- | --- | --- |
| DEG1001 | *Bacillus subtilis* 168 | 0.939 | 0.754 | 0.84 | 0.898 | 0.962 | 0.774 | 0.957 |
| DEG1002 | *Staphylococcus aureus* N315 | - | 0.822 | 0.9 | 0.873 | 0.829 | 0.799 | - |
| DEG1003 | *Vibrio cholerae* N16961 | 0.783 | 0.653 | 0.72 | 0.695 | - | 0.544 | 0.734 |
| DEG1005 | *Haemophilus influenzae* Rd KW20 | 0.558 | 0.534 | 0.77 | 0.566 | - | 0.553 | 0.572 |
| DEG1006 | *Mycoplasma genitalium* G37 | 0.672 | 0.554 | 0.66 | 0.631 | 0.713 | 0.690 | 0.690 |
| DEG1007 | *Streptococcus pneumoniae* | 0.764 | 0.704 | - | 0.743 | 0.721 | 0.651 | - |
| DEG1008 | *Helicobacter pylori* 26695 | 0.597 | 0.531 | 0.74 | 0.617 | 0.613 | 0.519 | 0.623 |
| DEG1011 | *Salmonella typhimurium* LT2 | - | 0.692 | 0.79 | 0.698 | 0.71 | 0.626 | - |
| DEG1012 | *Francisella novicida* U112 | 0.806 | 0.654 | 0.83 | 0.785 | 0.858 | 0.645 | 0.878 |
| DEG1013 | *Acinetobacter baylyi* ADP1 | 0.847 | - | 0.83 | 0.846 | 0.897 | 0.714 | 0.902 |
| DEG1014 | *Mycoplasma pulmonis* UAB CTIP | 0.868 | 0.650 | 0.74 | 0.819 | 0.861 | 0.708 | 0.849 |
| DEG1015 | *Pseudomonas aeruginosa* UCBPP-PA14 | 0.793 | 0.652 | 0.8 | 0.797 | 0.814 | 0.638 | - |
| DEG1017 | *Staphylococcus aureus* NCTC 8325 | 0.885 | 0.786 | 0.85 | 0.865 | 0.885 | 0.735 | 0.887 |
| DEG1019 | *Escherichia coli* MG1655 II | 0.947 | 0.861 | 0.88 | 0.908 | 0.956 | 0.706 | 0.940 |
| DEG1020 | *Caulobacter crescentus* | 0.901 | 0.740 | - | 0.885 | 0.932 | 0.693 | 0.929 |
| DEG1021 | *Streptococcus sanguinis* | 0.927 | 0.816 | - | 0.917 | 0.971 | 0.771 | 0.958 |
| DEG1023 | *Bacteroides thetaiotaomicron* VPI-5482 | 0.763 | 0.750 | - | 0.721 | 0.796 | 0.669 | 0.775 |
| DEG1024 | *Burkholderia thailandensis* E264 | - | 0.777 | - | 0.928 | 0.926 | 0.720 | 0.936 |
| DEG1026 | *Salmonella enterica* subsp. *enterica* serovar Typhimurium str. 14028S | - | 0.591 | - | - | 0.654 | 0.630 | - |
| DEG1027 | *Mycobacterium tuberculosis* H37Rv III | 0.787 | 0.666 | 0.77 | 0.764 | 0.759 | 0.607 | 0.663 |
| DEG1028 | *Sphingomonas wittichii* RW1 | - | 0.672 | - | 0.829 | 0.823 | 0.631 | 0.828 |
| DEG1029 | *Shewanella oneidensis* MR-1 | - | 0.781 | - | 0.907 | 0.912 | 0.739 | - |
| DEG1032 | *Salmonella enterica* serovar Typhimurium SL1344 | - | 0.831 | - | - | 0.886 | 0.728 | 0.946 |
| DEG1033 | *Salmonella enterica* serovar Typhi Ty2 | 0.924 | 0.901 | 0.86 | 0.930 | 0.961 | 0.724 | 0.970 |
| DEG1034 | *Bacteroides fragilis* 638R | - | 0.667 | - | - | 0.735 | 0.636 | 0.731 |
| DEG1035 | *Burkholderia pseudomallei* K96243 | - | 0.674 | - | 0.698 | 0.736 | 0.574 | 0.707 |
| DEG1036 | *Pseudomonas aeruginosa* PAO1 | - | 0.580 | - | 0.715 | 0.945 | 0.538 | 0.917 |
| DEG1037 | *Streptococcus pyogenes* MGAS5448 | - | 0.882 | - | - | 0.943 | 0.670 | - |
| DEG1038 | *Streptococcus pyogenes* NZ131 | - | 0.882 | - | 0.834 | 0.923 | 0.671 | 0.912 |
| DEG1039 | *Porphyromonas gingivalis* ATCC 33277 | 0.827 | 0.695 | - | 0.755 | 0.835 | 0.672 | 0.800 |
| DEG1040 | *Synechococcus elongatus* PCC 7942 | - | - | - | - | 0.772 | 0.684 | 0.798 |
| DEG1041 | *Rhodopseudomonas palustris* CGA009 | - | - | - | - | 0.903 | 0.666 | 0.910 |
| DEG1042 | *Streptococcus agalactiae* A909 | - | - | - | - | 0.905 | 0.724 | 0.897 |
| DEG1043 | *Acinetobacter baumannii* ATCC 17978 | - | 0.668 | - | - | 0.838 | 0.654 | 0.848 |
| DEG1045 | *Agrobacterium fabrum* C58 | - | - | - | - | 0.905 | 0.719 | 0.914 |
| DEG1046 | *Brevundimonas subvibrioides* ATCC 15264 | - | - | - | - | 0.944 | 0.718 | 0.953 |
| DEG1047 | *Bacillus thuringiensis* BMB171 | - | - | - | - | - | 0.545 | - |
| DEG1049 | *Campylobacter jejuni* subsp. *jejuni* NCTC 11168 = ATCC 700819 | - | 0.576 | - | 0.652 | 0.703 | 0.490 | - |
| DEG1050 | *Campylobacter jejuni* subsp. *jejuni* 81-176 | - | - | - | - | 0.749 | 0.576 | 0.721 |
| **AVERAGE** |  | 0.803  ± 0.113 | 0.708  ± 0.106 | 0.796  ± 0.066 | 0.784  ± 0.105 | 0.838  ± 0.098 | 0.657  ± 0.075 | 0.834  ± 0.112 |

**Supplementary Table S6**. Genes from *B. quintana*’s genome classified as essential by DELEAT. Their presence in the Core Minimal Genome proposed by Gil *et al*. (2004) is indicated.

| **locus_tag** | **Function** | **in CMG** |
| --- | --- | --- |
| BQ_RS00015 | shikimate dehydrogenase |  |
| BQ_RS00020 | dephospho-CoA kinase |  |
| BQ_RS00025 | DNA polymerase III subunit epsilon | dnaQ |
| BQ_RS00030 | DNA polymerase I | polA |
| BQ_RS00045 | lipoprotein signal peptidase |  |
| BQ_RS00080 | LPS export ABC transporter ATP-binding protein |  |
| BQ_RS00120 | thioredoxin |  |
| BQ_RS00140 | bifunctional tRNA (adenosine(37)-N6)-threonylcarbamoyltransferase complex ATPase subunit type 1 TsaE/phosphotransferase |  |
| BQ_RS00160 | bifunctional folylpolyglutamate synthase/dihydrofolate synthase |  |
| BQ_RS00165 | acetyl-CoA carboxylase carboxyltransferase subunit beta |  |
| BQ_RS00175 | bifunctional phosphopantothenoylcysteine decarboxylase/phosphopantothenate--cysteine ligase CoaBC |  |
| BQ_RS00180 | 2-polyprenylphenol 6-hydroxylase |  |
| BQ_RS00185 | bifunctional demethylmenaquinone methyltransferase/2-methoxy-6-polyprenyl-1,4-benzoquinol methylase UbiE |  |
| BQ_RS00190 | DNA topoisomerase (ATP-hydrolyzing) subunit B | gyrB |
| BQ_RS00195 | hypothetical protein |  |
| BQ_RS00220 | ABC transporter ATP-binding protein |  |
| BQ_RS00225 | translation initiation factor IF-3 | infC |
| BQ_RS00240 | succinyl-diaminopimelate desuccinylase |  |
| BQ_RS00260 | nucleotide exchange factor GrpE | grpE |
| BQ_RS00280 | hypothetical protein |  |
| BQ_RS00290 | DNA-binding response regulator |  |
| BQ_RS00305 | molecular chaperone DnaK | dnaK |
| BQ_RS00330 | 2,3,4,5-tetrahydropyridine-2,6-dicarboxylate N-succinyltransferase |  |
| BQ_RS00345 | methionyl-tRNA formyltransferase |  |
| BQ_RS00350 | peptide deformylase |  |
| BQ_RS00385 | 50S ribosomal protein L35 | rpmI |
| BQ_RS00390 | 50S ribosomal protein L20 | rplT |
| BQ_RS00395 | phenylalanine--tRNA ligase subunit alpha | pheS |
| BQ_RS00400 | phenylalanine--tRNA ligase subunit beta | pheT |
| BQ_RS00440 | 30S ribosomal protein S1 |  |
| BQ_RS00445 | (d)CMP kinase |  |
| BQ_RS00460 | tRNA-Ala |  |
| BQ_RS00525 | DsbE family thiol:disulfide interchange protein |  |
| BQ_RS00550 | aconitate hydratase AcnA |  |
| BQ_RS00555 | 30S ribosomal protein S20 | rpsT |
| BQ_RS00560 | chromosomal replication initiator protein DnaA | dnaA |
| BQ_RS00565 | DNA polymerase III subunit beta | dnaN |
| BQ_RS00575 | type II 3-dehydroquinate dehydratase |  |
| BQ_RS00600 | glycerol-3-phosphate dehydrogenase (NAD(P)(+)) |  |
| BQ_RS00640 | tRNA-Arg | tRNA-Arg |
| BQ_RS00655 | glucose-6-phosphate isomerase |  |
| BQ_RS00660 | tRNA-Ser | tRNA-Ser |
| BQ_RS00665 | 50S ribosomal protein L21 | rplU |
| BQ_RS00670 | 50S ribosomal protein L27 | rpmA |
| BQ_RS00680 | tRNA-Ser | tRNA-Ser |
| BQ_RS00730 | GTPase ObgE | obg |
| BQ_RS00745 | nicotinate-nucleotide adenylyltransferase |  |
| BQ_RS00780 | YggT family protein |  |
| BQ_RS00790 | inorganic diphosphatase |  |
| BQ_RS00800 | hypothetical protein |  |
| BQ_RS00825 | UTP--glucose-1-phosphate uridylyltransferase |  |
| BQ_RS00850 | IMP dehydrogenase |  |
| BQ_RS00860 | glutamine-hydrolyzing GMP synthase |  |
| BQ_RS00910 | class Ib ribonucleoside-diphosphate reductase assembly flavoprotein NrdI |  |
| BQ_RS00915 | class 1b ribonucleoside-diphosphate reductase subunit alpha |  |
| BQ_RS00920 | class 1b ribonucleoside-diphosphate reductase subunit beta |  |
| BQ_RS00925 | peptide chain release factor N(5)-glutamine methyltransferase | hemK |
| BQ_RS00930 | peptide chain release factor 1 | prfA |
| BQ_RS00935 | transcriptional repressor |  |
| BQ_RS00940 | preprotein translocase subunit SecA | secA |
| BQ_RS00970 | type I pantothenate kinase |  |
| BQ_RS00995 | polyribonucleotide nucleotidyltransferase | pnp |
| BQ_RS01000 | 30S ribosomal protein S15 | rpsO |
| BQ_RS01005 | hypothetical protein |  |
| BQ_RS01020 | 30S ribosome-binding factor RbfA | rbfA |
| BQ_RS01025 | translation initiation factor IF-2 | infB |
| BQ_RS01035 | transcription termination/antitermination protein NusA | nusA |
| BQ_RS01040 | ribosome maturation factor |  |
| BQ_RS01050 | methionine adenosyltransferase |  |
| BQ_RS01060 | apolipoprotein N-acyltransferase |  |
| BQ_RS01070 | rRNA maturation RNase YbeY | ybeY |
| BQ_RS01090 | tRNA (adenosine(37)-N6)-threonylcarbamoyltransferase complex dimerization subunit type 1 TsaB |  |
| BQ_RS01100 | YbaB/EbfC family nucleoid-associated protein |  |
| BQ_RS01105 | DNA polymerase III subunit gamma/tau | dnaX |
| BQ_RS06855 | signal recognition particle sRNA small type | ffs |
| BQ_RS01110 | hypothetical protein |  |
| BQ_RS01120 | 3-deoxy-manno-octulosonate cytidylyltransferase |  |
| BQ_RS01175 | phosphate signaling complex protein PhoU |  |
| BQ_RS01205 | hypothetical protein |  |
| BQ_RS01230 | isoleucine--tRNA ligase | ileS |
| BQ_RS01240 | nucleoside deaminase |  |
| BQ_RS01270 | DUF4170 domain-containing protein |  |
| BQ_RS01275 | 3-deoxy-D-manno-octulosonic acid transferase |  |
| BQ_RS01280 | tetraacyldisaccharide 4'-kinase |  |
| BQ_RS01335 | tRNA-Thr | tRNA-Thr |
| BQ_RS01345 | cobalt ABC transporter ATP-binding protein |  |
| BQ_RS01350 | energy-coupling factor transporter transmembrane protein EcfT |  |
| BQ_RS06870 | hypothetical protein |  |
| BQ_RS01365 | hypothetical protein |  |
| BQ_RS01380 | tRNA-Phe | tRNA-Phe |
| BQ_RS01390 | adenylosuccinate synthetase |  |
| BQ_RS01400 | RNA polymerase sigma factor RpoH |  |
| BQ_RS01440 | tRNA-His | tRNA-His |
| BQ_RS01470 | bifunctional methylenetetrahydrofolate dehydrogenase/methenyltetrahydrofolate cyclohydrolase |  |
| BQ_RS01490 | hypothetical protein |  |
| BQ_RS01515 | bifunctional 3-demethylubiquinone 3-O-methyltransferase/2-octaprenyl-6-hydroxy phenol methylase |  |
| BQ_RS01520 | aspartate kinase |  |
| BQ_RS01525 | hypothetical protein |  |
| BQ_RS01550 | ribosome biogenesis GTPase Der | engA |
| BQ_RS01560 | F0F1 ATP synthase subunit A | atpB |
| BQ_RS01565 | F0F1 ATP synthase subunit C | atpE |
| BQ_RS01570 | ATP synthase subunit b 1 | atpF |
| BQ_RS01580 | glycine--tRNA ligase subunit beta | glyS |
| BQ_RS01585 | glycine--tRNA ligase subunit alpha |  |
| BQ_RS01595 | polyprenyl synthetase family protein |  |
| BQ_RS01610 | 4-diphosphocytidyl-2C-methyl-D-erythritol kinase |  |
| BQ_RS07115 | ubiquinol-cytochrome-c reductase |  |
| BQ_RS01640 | aminoacyl-tRNA hydrolase | pth |
| BQ_RS01645 | 50S ribosomal protein L25 |  |
| BQ_RS01665 | ribose-phosphate pyrophosphokinase |  |
| BQ_RS01670 | hypothetical protein |  |
| BQ_RS01680 | ATP synthase subunit beta | atpD |
| BQ_RS01685 | prolipoprotein diacylglyceryl transferase |  |
| BQ_RS01705 | response regulator |  |
| BQ_RS01730 | threonylcarbamoyl-AMP synthase |  |
| BQ_RS01750 | 4-hydroxybenzoate octaprenyltransferase |  |
| BQ_RS01775 | 3,4-dihydroxy-2-butanone-4-phosphate synthase |  |
| BQ_RS01785 | 1-deoxy-D-xylulose-5-phosphate synthase |  |
| BQ_RS01820 | 4-hydroxy-3-methylbut-2-enyl diphosphate reductase |  |
| BQ_RS01855 | hypothetical protein |  |
| BQ_RS01915 | peptidoglycan-binding protein |  |
| BQ_RS01930 | SufE family protein |  |
| BQ_RS01945 | superoxide dismutase |  |
| BQ_RS01950 | tRNA-Gln | tRNA-Gln |
| BQ_RS02070 | thiamine phosphate synthase |  |
| BQ_RS02080 | sulfur carrier protein ThiS |  |
| BQ_RS02130 | 4-hydroxy-tetrahydrodipicolinate synthase |  |
| BQ_RS02135 | SsrA-binding protein | smpB |
| BQ_RS02150 | bifunctional (p)ppGpp synthetase/guanosine-3',5'-bis(diphosphate) 3'-pyrophosphohydrolase |  |
| BQ_RS02165 | holo-ACP synthase |  |
| BQ_RS02170 | signal peptidase I | lepB |
| BQ_RS02180 | GTPase Era | era |
| BQ_RS02190 | pantoate--beta-alanine ligase |  |
| BQ_RS02205 | folate-binding protein |  |
| BQ_RS02215 | TIGR02301 family protein |  |
| BQ_RS02220 | cysteine--tRNA ligase | cysS |
| BQ_RS02235 | phosphatidylserine decarboxylase |  |
| BQ_RS02240 | CDP-diacylglycerol--serine O-phosphatidyltransferase |  |
| BQ_RS02265 | replicative DNA helicase | dnaB |
| BQ_RS02280 | 30S ribosomal protein S18 | rpsR |
| BQ_RS02285 | 30S ribosomal protein S6 | rpsF |
| BQ_RS02295 | [acyl-carrier-protein] S-malonyltransferase |  |
| BQ_RS02300 | 3-oxoacyl-[acyl-carrier-protein] reductase |  |
| BQ_RS02305 | acyl carrier protein |  |
| BQ_RS02310 | beta-ketoacyl-[acyl-carrier-protein] synthase II |  |
| BQ_RS02320 | guanylate kinase |  |
| BQ_RS02330 | 4-hydroxythreonine-4-phosphate dehydrogenase PdxA |  |
| BQ_RS02340 | LPS-assembly protein LptD |  |
| BQ_RS02345 | LPS export ABC transporter permease LptG |  |
| BQ_RS02350 | LPS export ABC transporter permease LptF |  |
| BQ_RS02380 | nucleoside-diphosphate kinase |  |
| BQ_RS02385 | CDP-diacylglycerol--glycerol-3-phosphate 3-phosphatidyltransferase |  |
| BQ_RS02410 | hypothetical protein |  |
| BQ_RS02415 | twin-arginine translocase subunit TatC |  |
| BQ_RS02420 | serine--tRNA ligase | serS |
| BQ_RS02435 | preprotein translocase subunit YajC |  |
| BQ_RS02455 | triose-phosphate isomerase |  |
| BQ_RS02465 | CTP synthetase |  |
| BQ_RS02470 | 3-deoxy-8-phosphooctulonate synthase |  |
| BQ_RS02475 | enolase |  |
| BQ_RS02510 | lipoyl synthase |  |
| BQ_RS02515 | type II toxin-antitoxin system RatA family toxin |  |
| BQ_RS02525 | bifunctional 2-C-methyl-D-erythritol 4-phosphate cytidylyltransferase/2-C-methyl-D-erythritol 2,4-cyclodiphosphate synthase |  |
| BQ_RS02555 | ATP-dependent Clp protease proteolytic subunit |  |
| BQ_RS02565 | endopeptidase La | lon |
| BQ_RS02580 | tRNA-Leu | tRNA-Leu |
| BQ_RS02590 | hypothetical protein |  |
| BQ_RS02595 | tRNA-Ser | tRNA-Ser |
| BQ_RS02610 | tRNA-Val | tRNA-Val |
| BQ_RS02620 | lysophospholipase |  |
| BQ_RS02630 | tRNA-Pro | tRNA-Pro |
| BQ_RS02645 | lipid A biosynthesis lauroyl acyltransferase |  |
| BQ_RS02675 | hypothetical protein |  |
| BQ_RS02680 | hypothetical protein |  |
| BQ_RS02705 | N-acetyl-gamma-glutamyl-phosphate reductase |  |
| BQ_RS02710 | serine hydroxymethyltransferase |  |
| BQ_RS02720 | bifunctional diaminohydroxyphosphoribosylaminopyrimidine deaminase/5-amino-6-(5-phosphoribosylamino)uracil reductase RibD |  |
| BQ_RS02725 | riboflavin synthase |  |
| BQ_RS02730 | 6,7-dimethyl-8-ribityllumazine synthase |  |
| BQ_RS02735 | N utilization substance protein B |  |
| BQ_RS02740 | outer membrane protein assembly factor BamE |  |
| BQ_RS02750 | phosphate acyltransferase |  |
| BQ_RS02755 | 3-oxoacyl-ACP synthase III |  |
| BQ_RS02770 | threonine--tRNA ligase | thrS |
| BQ_RS02785 | GTP cyclohydrolase I FolE |  |
| BQ_RS02790 | tRNA-Leu | tRNA-Leu |
| BQ_RS02800 | hypothetical protein |  |
| BQ_RS02820 | tRNA-Val | tRNA-Val |
| BQ_RS02825 | tRNA-Asp | tRNA-Asp |
| BQ_RS02830 | NADH-quinone oxidoreductase subunit A |  |
| BQ_RS02835 | NADH-quinone oxidoreductase subunit B |  |
| BQ_RS02840 | NADH-quinone oxidoreductase subunit C |  |
| BQ_RS02845 | NADH-quinone oxidoreductase subunit D |  |
| BQ_RS02850 | NADH-quinone oxidoreductase subunit NuoE |  |
| BQ_RS02860 | NADH-quinone oxidoreductase subunit G |  |
| BQ_RS02865 | NADH-quinone oxidoreductase subunit NuoH |  |
| BQ_RS02870 | NADH-quinone oxidoreductase subunit NuoI |  |
| BQ_RS02875 | NADH-quinone oxidoreductase subunit J |  |
| BQ_RS02880 | NADH-quinone oxidoreductase subunit K |  |
| BQ_RS02885 | NADH-quinone oxidoreductase subunit L |  |
| BQ_RS02890 | NADH-quinone oxidoreductase subunit M |  |
| BQ_RS02895 | NADH-quinone oxidoreductase subunit NuoN |  |
| BQ_RS02900 | biotin--[acetyl-CoA-carboxylase] ligase |  |
| BQ_RS02905 | MBL fold metallo-hydrolase |  |
| BQ_RS02910 | proline--tRNA ligase | proS |
| BQ_RS02915 | lipoprotein-releasing ABC transporter permease subunit |  |
| BQ_RS02920 | ABC transporter ATP-binding protein |  |
| BQ_RS02945 | ribonuclease E/G |  |
| BQ_RS02950 | N-acetylmuramoyl-L-alanine amidase |  |
| BQ_RS02960 | peptide chain release factor 2 |  |
| BQ_RS02970 | tyrosine--tRNA ligase | tyrS |
| BQ_RS02980 | cysteine desulfurase | iscS |
| BQ_RS02985 | Fe-S cluster assembly protein SufB |  |
| BQ_RS02990 | Fe-S cluster assembly ATPase SufC |  |
| BQ_RS02995 | Fe-S cluster assembly protein SufD |  |
| BQ_RS03000 | cysteine desulfurase | iscS |
| BQ_RS03015 | hypothetical protein |  |
| BQ_RS03045 | methionine--tRNA ligase | metS |
| BQ_RS03050 | DNA polymerase III subunit delta' | holB |
| BQ_RS03055 | dTMP kinase |  |
| BQ_RS03070 | tRNA-Lys | tRNA-Lys |
| BQ_RS03075 | transcriptional repressor LexA |  |
| BQ_RS03090 | 30S ribosomal protein S4 | rpsD |
| BQ_RS03095 | glutamate racemase |  |
| BQ_RS03110 | type I methionyl aminopeptidase | map |
| BQ_RS03120 | DUF3126 family protein |  |
| BQ_RS03165 | Asp-tRNA(Asn)/Glu-tRNA(Gln) amidotransferase subunit GatA |  |
| BQ_RS03170 | Asp-tRNA(Asn)/Glu-tRNA(Gln) amidotransferase subunit GatC |  |
| BQ_RS03175 | Holliday junction resolvase RuvX |  |
| BQ_RS03195 | glycerol-3-phosphate 1-O-acyltransferase |  |
| BQ_RS03205 | type I DNA topoisomerase |  |
| BQ_RS03215 | 50S ribosomal protein L33 | rpmG |
| BQ_RS06955 | hypothetical protein |  |
| BQ_RS03250 | acetyl-CoA carboxylase biotin carboxyl carrier protein |  |
| BQ_RS03255 | acetyl-CoA carboxylase biotin carboxylase subunit |  |
| BQ_RS03260 | tRNA-Thr | tRNA-Thr |
| BQ_RS03285 | lipoate-protein ligase B |  |
| BQ_RS03290 | DNA topoisomerase IV subunit B |  |
| BQ_RS03300 | 50S ribosomal protein L13 | rplM |
| BQ_RS03305 | 30S ribosomal protein S9 | rpsI |
| BQ_RS03320 | hypothetical protein |  |
| BQ_RS03335 | hypothetical protein |  |
| BQ_RS03345 | tRNA-Arg | tRNA-Arg |
| BQ_RS03350 | NAD kinase |  |
| BQ_RS03365 | NAD+ synthase |  |
| BQ_RS03385 | ribose-5-phosphate isomerase |  |
| BQ_RS03395 | glutamate--tRNA ligase | gltX |
| BQ_RS03400 | citrate synthase |  |
| BQ_RS03420 | amino acid ABC transporter ATP-binding protein |  |
| BQ_RS03425 | lipid-A-disaccharide synthase |  |
| BQ_RS03435 | acyl-[acyl-carrier-protein]--UDP-N- acetylglucosamine O-acyltransferase |  |
| BQ_RS03440 | 3-hydroxyacyl-ACP dehydratase FabZ |  |
| BQ_RS03445 | UDP-3-O-(3-hydroxymyristoyl)glucosamine N-acyltransferase |  |
| BQ_RS03450 | outer membrane protein assembly factor BamA |  |
| BQ_RS03455 | RIP metalloprotease RseP |  |
| BQ_RS03460 | phosphatidate cytidylyltransferase |  |
| BQ_RS03465 | ribosome-recycling factor |  |
| BQ_RS03470 | UMP kinase |  |
| BQ_RS03475 | elongation factor Ts | tsf |
| BQ_RS03480 | 30S ribosomal protein S2 | rpsB |
| BQ_RS03505 | ATP-dependent Clp protease adapter ClpS |  |
| BQ_RS03530 | DNA-directed RNA polymerase subunit beta' | rpoC |
| BQ_RS03535 | DNA-directed RNA polymerase subunit beta | rpoB |
| BQ_RS03540 | 50S ribosomal protein L7/L12 | rplL |
| BQ_RS03545 | 50S ribosomal protein L10 | rplJ |
| BQ_RS03550 | 50S ribosomal protein L1 | rplA |
| BQ_RS03555 | 50S ribosomal protein L11 | rplK |
| BQ_RS03560 | transcription termination/antitermination protein NusG | nusG |
| BQ_RS03565 | preprotein translocase subunit SecE | secE |
| BQ_RS03570 | tRNA-Trp | tRNA-Trp |
| BQ_RS03580 | tRNA-Gly | tRNA-Gly |
| BQ_RS03585 | tRNA-Tyr | tRNA-Tyr |
| BQ_RS03610 | Asp-tRNA(Asn)/Glu-tRNA(Gln) amidotransferase subunit GatB |  |
| BQ_RS03625 | tRNA-Arg | tRNA-Arg |
| BQ_RS03635 | tRNA-Pro | tRNA-Pro |
| BQ_RS03640 | hypothetical protein |  |
| BQ_RS03645 | hypothetical protein |  |
| BQ_RS03665 | response regulator |  |
| BQ_RS03685 | ribulose-phosphate 3-epimerase |  |
| BQ_RS03690 | adenylosuccinate lyase |  |
| BQ_RS03710 | phosphoribosylformylglycinamidine synthase subunit PurQ |  |
| BQ_RS03725 | Grx4 family monothiol glutaredoxin |  |
| BQ_RS03735 | DNA topoisomerase IV subunit A |  |
| BQ_RS03740 | aspartate--tRNA(Asp/Asn) ligase | asnS, aspS |
| BQ_RS03770 | DNA polymerase III subunit alpha | dnaE |
| BQ_RS03780 | glutamine--fructose-6-phosphate transaminase (isomerizing) |  |
| BQ_RS03785 | bifunctional N-acetylglucosamine-1-phosphate uridyltransferase/glucosamine-1-phosphate acetyltransferase |  |
| BQ_RS03800 | iron-sulfur cluster assembly accessory protein |  |
| BQ_RS03810 | arginine--tRNA ligase | argS |
| BQ_RS03840 | tRNA-Glu | tRNA-Glu |
| BQ_RS03850 | NADP-dependent isocitrate dehydrogenase |  |
| BQ_RS03865 | hypothetical protein |  |
| BQ_RS03885 | phosphopantetheine adenylyltransferase |  |
| BQ_RS03890 | DNA gyrase subunit A | gyrA |
| BQ_RS03895 | single-stranded DNA-binding protein | ssb |
| BQ_RS03905 | P-II family nitrogen regulator |  |
| BQ_RS03910 | type I glutamate--ammonia ligase |  |
| BQ_RS03915 | hypothetical protein |  |
| BQ_RS03935 | alanine--tRNA ligase | alaS |
| BQ_RS03955 | 50S ribosomal protein L17 | rplQ |
| BQ_RS03960 | DNA-directed RNA polymerase subunit alpha | rpoA |
| BQ_RS03965 | 30S ribosomal protein S11 | rpsK |
| BQ_RS03970 | 30S ribosomal protein S13 | rpsM |
| BQ_RS03975 | adenylate kinase |  |
| BQ_RS03980 | preprotein translocase subunit SecY | secY |
| BQ_RS03985 | 50S ribosomal protein L15 | rplO |
| BQ_RS03990 | 50S ribosomal protein L30 |  |
| BQ_RS03995 | 30S ribosomal protein S5 | rpsE |
| BQ_RS04000 | 50S ribosomal protein L18 | rplR |
| BQ_RS04005 | 50S ribosomal protein L6 | rplF |
| BQ_RS04010 | 30S ribosomal protein S8 | rpsH |
| BQ_RS04015 | 30S ribosomal protein S14 | rpsN |
| BQ_RS04020 | 50S ribosomal protein L5 | rplE |
| BQ_RS04025 | 50S ribosomal protein L24 | rplX |
| BQ_RS04030 | 50S ribosomal protein L14 | rplN |
| BQ_RS04035 | 30S ribosomal protein S17 | rpsQ |
| BQ_RS04040 | 50S ribosomal protein L29 | rpmC |
| BQ_RS04045 | 50S ribosomal protein L16 | rplP |
| BQ_RS04050 | 30S ribosomal protein S3 | rpsC |
| BQ_RS04055 | 50S ribosomal protein L22 | rplV |
| BQ_RS04060 | 30S ribosomal protein S19 | rpsS |
| BQ_RS04065 | 50S ribosomal protein L2 | rplB |
| BQ_RS04070 | 50S ribosomal protein L23 | rplW |
| BQ_RS04075 | 50S ribosomal protein L4 | rplD |
| BQ_RS04080 | 50S ribosomal protein L3 | rplC |
| BQ_RS04085 | 30S ribosomal protein S10 | rpsJ |
| BQ_RS04095 | elongation factor G | fusA |
| BQ_RS04100 | 30S ribosomal protein S7 | rpsG |
| BQ_RS04105 | 30S ribosomal protein S12 | rpsL |
| BQ_RS04140 | valine--tRNA ligase | valS |
| BQ_RS04160 | tRNA-Cys | tRNA-Cys |
| BQ_RS04165 | tRNA-Asn | tRNA-Asn |
| BQ_RS04175 | tRNA-Leu | tRNA-Leu |
| BQ_RS04195 | hypothetical protein |  |
| BQ_RS04200 | protein translocase subunit SecD |  |
| BQ_RS04220 | tRNA-Lys | tRNA-Lys |
| BQ_RS04230 | hypothetical protein |  |
| BQ_RS04240 | ketol-acid reductoisomerase |  |
| BQ_RS04250 | pyridoxine 5'-phosphate synthase |  |
| BQ_RS04265 | tRNA (adenosine(37)-N6)-dimethylallyltransferase MiaA |  |
| BQ_RS04285 | dihydrofolate reductase |  |
| BQ_RS04290 | thymidylate synthase |  |
| BQ_RS07000 | transfer-messenger RNA | ssrA |
| BQ_RS04325 | ATP-dependent DNA helicase | deaD |
| BQ_RS04330 | SCO family protein |  |
| BQ_RS04345 | NAD-dependent DNA ligase LigA | lig |
| BQ_RS04355 | outer membrane protein assembly factor BamD |  |
| BQ_RS04360 | UDP-3-O-acyl-N-acetylglucosamine deacetylase |  |
| BQ_RS04365 | cell division protein FtsZ | ftsZ |
| BQ_RS04370 | cell division protein FtsA |  |
| BQ_RS04375 | cell division protein FtsQ/DivIB |  |
| BQ_RS04380 | D-alanine--D-alanine ligase |  |
| BQ_RS04385 | UDP-N-acetylmuramate dehydrogenase |  |
| BQ_RS04390 | UDP-N-acetylmuramate--L-alanine ligase |  |
| BQ_RS04395 | undecaprenyldiphospho-muramoylpentapeptide beta-N-acetylglucosaminyltransferase |  |
| BQ_RS04400 | cell division protein FtsW |  |
| BQ_RS04405 | UDP-N-acetylmuramoyl-L-alanine--D-glutamate ligase |  |
| BQ_RS04410 | phospho-N-acetylmuramoyl-pentapeptide- transferase |  |
| BQ_RS04415 | UDP-N-acetylmuramoyl-tripeptide--D-alanyl-D- alanine ligase |  |
| BQ_RS04420 | UDP-N-acetylmuramoyl-L-alanyl-D-glutamate--2, 6-diaminopimelate ligase |  |
| BQ_RS04425 | penicillin-binding protein 2 |  |
| BQ_RS04435 | ribosomal RNA small subunit methyltransferase H | mraW |
| BQ_RS06825 | RNase P RNA component class A | rnpB |
| BQ_RS04480 | XRE family transcriptional regulator |  |
| BQ_RS07020 | hypothetical protein |  |
| BQ_RS04505 | tRNA-Met | tRNA-Met |
| BQ_RS04520 | RNA polymerase sigma factor RpoD | rpoD |
| BQ_RS04530 | DNA primase | dnaG |
| BQ_RS04555 | carbamoyl-phosphate synthase small subunit |  |
| BQ_RS04585 | thioredoxin-disulfide reductase |  |
| BQ_RS04630 | CCA tRNA nucleotidyltransferase |  |
| BQ_RS04670 | tRNA 2-thiouridine(34) synthase MnmA | mnmA |
| BQ_RS04715 | tRNA-Met | tRNA-Met |
| BQ_RS04775 | electron transfer flavoprotein subunit beta/FixA family protein |  |
| BQ_RS04820 | 50S ribosomal protein L34 | rpmH |
| BQ_RS04825 | ribonuclease P protein component | rnpA |
| BQ_RS04830 | membrane protein insertase YidC | yidC |
| BQ_RS04835 | YihA family ribosome biogenesis GTP-binding protein |  |
| BQ_RS04845 | 4-hydroxy-tetrahydrodipicolinate reductase |  |
| BQ_RS04850 | 2,3-bisphosphoglycerate-dependent phosphoglycerate mutase |  |
| BQ_RS04855 | tRNA-Leu | tRNA-Leu |
| BQ_RS04875 | tRNA-Ser | tRNA-Ser |
| BQ_RS04905 | tRNA-Met | tRNA-Met |
| BQ_RS04910 | 5S ribosomal RNA | rrfA |
| BQ_RS04915 | 23S ribosomal RNA | rrlA |
| BQ_RS04925 | tRNA-Ala | tRNA-Ala |
| BQ_RS04930 | tRNA-Ile | tRNA-Ile |
| BQ_RS04935 | 16S ribosomal RNA | rrsA |
| BQ_RS04980 | ferredoxin--NADP reductase |  |
| BQ_RS04985 | alanine racemase |  |
| BQ_RS05025 | aspartate-semialdehyde dehydrogenase |  |
| BQ_RS05115 | tRNA-Gln | tRNA-Gln |
| BQ_RS05180 | bifunctional riboflavin kinase/FAD synthetase |  |
| BQ_RS05200 | hypothetical protein |  |
| BQ_RS05225 | hypothetical protein |  |
| BQ_RS05305 | murein biosynthesis integral membrane protein MurJ |  |
| BQ_RS05310 | tryptophan--tRNA ligase | trpS |
| BQ_RS05330 | molecular chaperone GroEL | groEL |
| BQ_RS05335 | molecular chaperone GroES | groES |
| BQ_RS05360 | histidine--tRNA ligase | hisS |
| BQ_RS05380 | tRNA-Met | tRNA-Met |
| BQ_RS05385 | 5S ribosomal RNA | rrfA |
| BQ_RS05390 | 23S ribosomal RNA | rrlA |
| BQ_RS05400 | tRNA-Ala | tRNA-Ala |
| BQ_RS05405 | tRNA-Ile | tRNA-Ile |
| BQ_RS05410 | 16S ribosomal RNA | rrsA |
| BQ_RS05460 | hypothetical protein |  |
| BQ_RS05575 | translation initiation factor IF-1 | infA |
| BQ_RS07065 | 6S RNA |  |
| BQ_RS05645 | tRNA-Thr | tRNA-Thr |
| BQ_RS05650 | UDP-N-acetylglucosamine 1-carboxyvinyltransferase |  |
| BQ_RS05775 | tRNA-Leu | tRNA-Leu |
| BQ_RS05830 | phosphoglucosamine mutase |  |
| BQ_RS05835 | ATP-dependent metallopeptidase FtsH/Yme1/Tma family protein |  |
| BQ_RS05840 | tRNA lysidine(34) synthetase TilS | tilS |
| BQ_RS05885 | protein TolQ |  |
| BQ_RS05935 | elongation factor P | efp |
| BQ_RS05955 | 50S ribosomal protein L31 | rpmE |
| BQ_RS05965 | phosphoglycerate kinase |  |
| BQ_RS05970 | type I glyceraldehyde-3-phosphate dehydrogenase |  |
| BQ_RS05975 | transketolase |  |
| BQ_RS06020 | hypothetical protein |  |
| BQ_RS06035 | ferredoxin family protein |  |
| BQ_RS06055 | 4-hydroxy-3-methylbut-2-en-1-yl diphosphate synthase (flavodoxin) |  |
| BQ_RS06080 | ATP synthase subunit beta | atpD |
| BQ_RS06085 | ATP synthase subunit gamma | atpG |
| BQ_RS06090 | F0F1 ATP synthase subunit alpha | atpA |
| BQ_RS06095 | F0F1 ATP synthase subunit delta | atpH |
| BQ_RS06125 | leucine--tRNA ligase | leuS |
| BQ_RS06185 | ubiquinone biosynthesis hydroxylase |  |
| BQ_RS06195 | hypothetical protein |  |
| BQ_RS06245 | hypothetical protein |  |
| BQ_RS06310 | hypothetical protein |  |
| BQ_RS06330 | succinate dehydrogenase, hydrophobic membrane anchor protein |  |
| BQ_RS06340 | 50S ribosomal protein L19 | rplS |
| BQ_RS06345 | tRNA (guanosine(37)-N1)-methyltransferase TrmD | trmD |
| BQ_RS06350 | ribosome maturation factor RimM |  |
| BQ_RS06385 | 50S ribosomal protein L28 | rpmB |
| BQ_RS06420 | 30S ribosomal protein S16 | rpsP |
| BQ_RS06430 | signal recognition particle protein | ffh |
| BQ_RS06465 | tRNA (adenosine(37)-N6)-threonylcarbamoyltransferase complex transferase subunit TsaD | gcp |
| BQ_RS06485 | DNA-binding response regulator |  |
| BQ_RS06560 | tRNA-Gly | tRNA-Gly |
| BQ_RS06570 | 1-acyl-sn-glycerol-3-phosphate acyltransferase |  |
| BQ_RS06575 | hypothetical protein |  |
| BQ_RS06595 | acetyl-CoA carboxylase carboxyltransferase subunit alpha |  |
| BQ_RS06610 | shikimate kinase |  |
| BQ_RS06640 | Na+/H+ antiporter subunit E |  |
| BQ_RS06675 | dihydrolipoyl dehydrogenase |  |
| BQ_RS06680 | 2-oxoglutarate dehydrogenase complex dihydrolipoyllysine-residue succinyltransferase |  |
| BQ_RS06685 | 2-oxoglutarate dehydrogenase E1 component |  |
| BQ_RS06715 | diaminopimelate epimerase |  |
| BQ_RS06725 | signal recognition particle-docking protein FtsY | ftsY |
| BQ_RS06730 | septation protein A |  |
| BQ_RS06740 | ParB/RepB/Spo0J family partition protein |  |
| BQ_RS06745 | ParA family protein |  |
| BQ_RS06750 | ribosomal RNA small subunit methyltransferase G |  |
| BQ_RS06755 | tRNA uridine-5-carboxymethylaminomethyl(34) synthesis enzyme MnmG | mnmG |
| BQ_RS06760 | tRNA uridine-5-carboxymethylaminomethyl(34) synthesis GTPase MnmE | mnmE |
| BQ_RS06765 | transcription termination factor Rho |  |
| **False negatives (in CMG but predicted as non-essential by DELEAT)**  ***: “putative non-essential” in CMG** | | |
| BQ_RS06540***** | DNA polymerase III subunit delta | holA |
| BQ_RS02570 | HU family DNA-binding protein | hupA |
| BQ_RS00380 | endonuclease III | nth |
| BQ_RS02060 | uracil-DNA glycosylase | ung |
| BQ_RS04600 | transcription elongation factor GreA | greA |
| BQ_RS00110 | lysine--tRNA ligase | lysS |
| BQ_RS02270***** | 50S ribosomal protein L9 | rplI |
| BQ_RS06045***** | 50S ribosomal protein L32 | rpmF |
| BQ_RS05615***** | 50S ribosomal protein L36 | rpmJ |
| BQ_RS00050 | RNA methyltransferase | cspR |
| BQ_RS02325***** | 16S rRNA (adenine(1518)-N(6)/adenine(1519)-N(6))-dimethyltransferase RsmA | ksgA |
| BQ_RS04805 | 16S rRNA (cytidine(1402)-2'-O)-methyltransferase | rsmI |
| BQ_RS01625 | redox-regulated ATPase YchF | ychF |
| BQ_RS00325 | elongation factor 4 | lepA |
| BQ_RS04090 | elongation factor Tu | tufA |
| BQ_RS02175 | ribonuclease 3 | rnc |
| BQ_RS00965 | leucyl aminopeptidase family protein | pepA |
| BQ_RS00310 | molecular chaperone DnaJ | dnaJ |
| BQ_RS06075 | F0F1 ATP synthase subunit epsilon | atpC |

**Supplementary Table S7**. Deletions in *B. quintana*’s genome proposed by DELEAT. Start and end coordinates, as well as length in bp and as a fraction of the total genome size, are included for each deletion. The last column details each deletion’s gene content, classified into pseudogenes, genes annotated as “hypothetical protein”, and genes with functional annotation.

|  | start | end | length | % of genome | contains  (pseudo/hypot/non-hypot) |
| --- | --- | --- | --- | --- | --- |
| D1 | 25667 | 33713 | 8047 | 0,509 | 0/1/2 |
| D2 | 52828 | 62076 | 9249 | 0,585 | 0/0/4 |
| D3 | 102472 | 117237 | 14766 | 0,934 | 0/0/7 |
| D4 | 122210 | 138851 | 16642 | 1,052 | 3/2/7 |
| D5 | 156744 | 164023 | 7280 | 0,460 | 1/1/5 |
| D6 | 176687 | 193830 | 17144 | 1,084 | 2/2/5 |
| D7 | 207011 | 214037 | 7027 | 0,444 | 1/0/3 |
| D8 | 226101 | 234802 | 8702 | 0,550 | 0/0/9 |
| D9 | 285370 | 296070 | 10701 | 0,677 | 3/0/7 |
| D10 | 324211 | 336094 | 11884 | 0,751 | 2/2/5 |
| D11 | 350840 | 360735 | 9896 | 0,626 | 0/1/6 |
| D12 | 451689 | 464948 | 13260 | 0,839 | 1/3/7 |
| D13 | 474186 | 503382 | 29197 | 1,846 | 0/3/18 |
| D14 | 507911 | 521136 | 13226 | 0,836 | 0/0/9 |
| D15 | 739517 | 747431 | 7915 | 0,501 | 0/0/8 |
| D16 | 890304 | 897744 | 7441 | 0,471 | 0/0/5 |
| D17 | 913181 | 921487 | 8307 | 0,525 | 1/1/3 |
| D18 | 1054940 | 1069795 | 14856 | 0,939 | 2/4/7 |
| D19 | 1088246 | 1097855 | 9610 | 0,608 | 0/0/5 |
| D20 | 1110679 | 1121189 | 10511 | 0,665 | 1/0/6 |
| D21 | 1130183 | 1145694 | 15512 | 0,981 | 1/1/10 |
| D22 | 1164216 | 1172722 | 8507 | 0,538 | 1/0/4 |
| D23 | 1179013 | 1187492 | 8480 | 0,536 | 2/1/6 |
| D24 | 1190486 | 1198669 | 8184 | 0,518 | 1/1/5 |
| D25 | 1200102 | 1223601 | 23500 | 1,486 | 5/4/9 |
| D26 | 1224076 | 1244779 | 20704 | 1,309 | 0/8/3 |
| D27 | 1249540 | 1272464 | 22925 | 1,450 | 2/8/10 |
| D28 | 1297727 | 1306536 | 8810 | 0,557 | 2/3/5 |
| D29 | 1307198 | 1329592 | 22395 | 1,416 | 1/13/8 |
| D30 | 1330212 | 1337946 | 7735 | 0,489 | 0/3/4 |
| D31 | 1344266 | 1367635 | 23370 | 1,478 | 1/18/5 |
| D32 | 1368121 | 1376445 | 8325 | 0,526 | 3/1/7 |
| D33 | 1382386 | 1392361 | 9976 | 0,631 | 2/3/5 |
| D34 | 1393476 | 1401335 | 7860 | 0,497 | 1/0/8 |
| D35 | 1411559 | 1419489 | 7931 | 0,502 | 1/1/7 |
| D36 | 1443962 | 1460119 | 16158 | 1,022 | 0/0/11 |
| D37 | 1463545 | 1478903 | 15359 | 0,971 | 0/4/18 |
| D38 | 1493803 | 1502467 | 8665 | 0,548 | 0/0/6 |
| D39 | 1505168 | 1515795 | 10628 | 0,672 | 1/2/3 |
| D40 | 1521140 | 1531658 | 10519 | 0,665 | 2/2/6 |
| D41 | 1548213 | 1556897 | 8685 | 0,549 | 0/0/6 |

**SUPPLEMENTARY FIGURES**

**
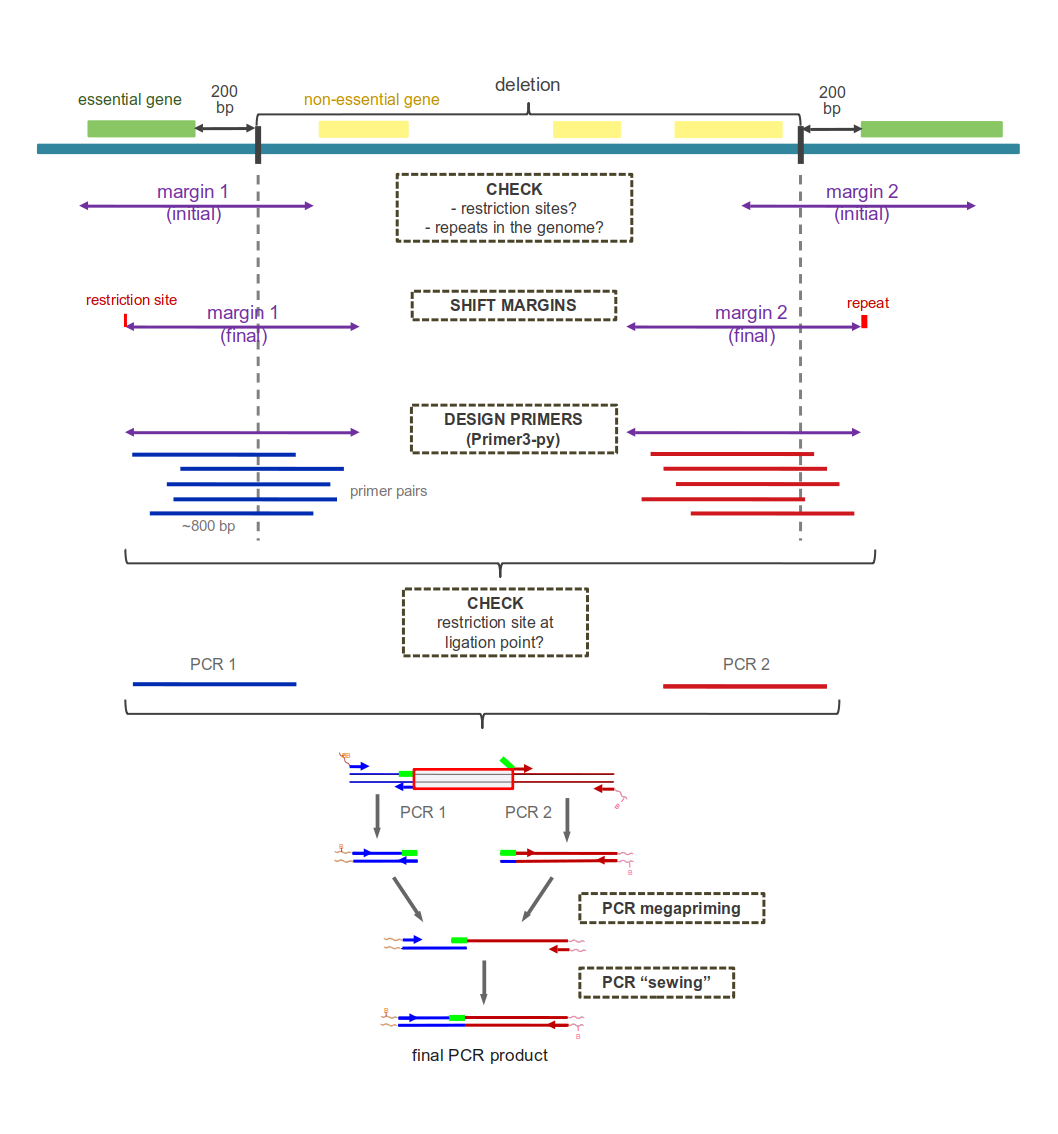
**

**Supplementary Figure S1**. Algorithm for PCR primer design in the DELEAT pipeline. Starting from a proposed deletion in the genome, a pair of 1000 bp-long “margins” is defined around the start and end coordinates, with 200 bp inside of the deletion and 800 outside. Next, two conditions are checked – that the margins do not contain neither target sites of the restriction enzyme selected for cloning, nor sequences longer than 20 bp that are repeated in the genome (in order to avoid undesired homologous recombination events). If any of these conditions is violated, the affected margin is shifted “inwards” (right in the case of margin 1 and left for 2) until the problematic position falls outside it. This process is repeated until both margins comply with the primer design rules. Once the final margin coordinates are obtained, functions from the Primer3-py package are used to generate a list of the best 20 primer pairs inside the margins. The best pair of PCR products is then decided as those generated from the best primer pairs and which do not form a restriction target site when concatenated, or have a size difference larger than 400 bp. These are the PCR products that will be used for the megapriming reaction.

| 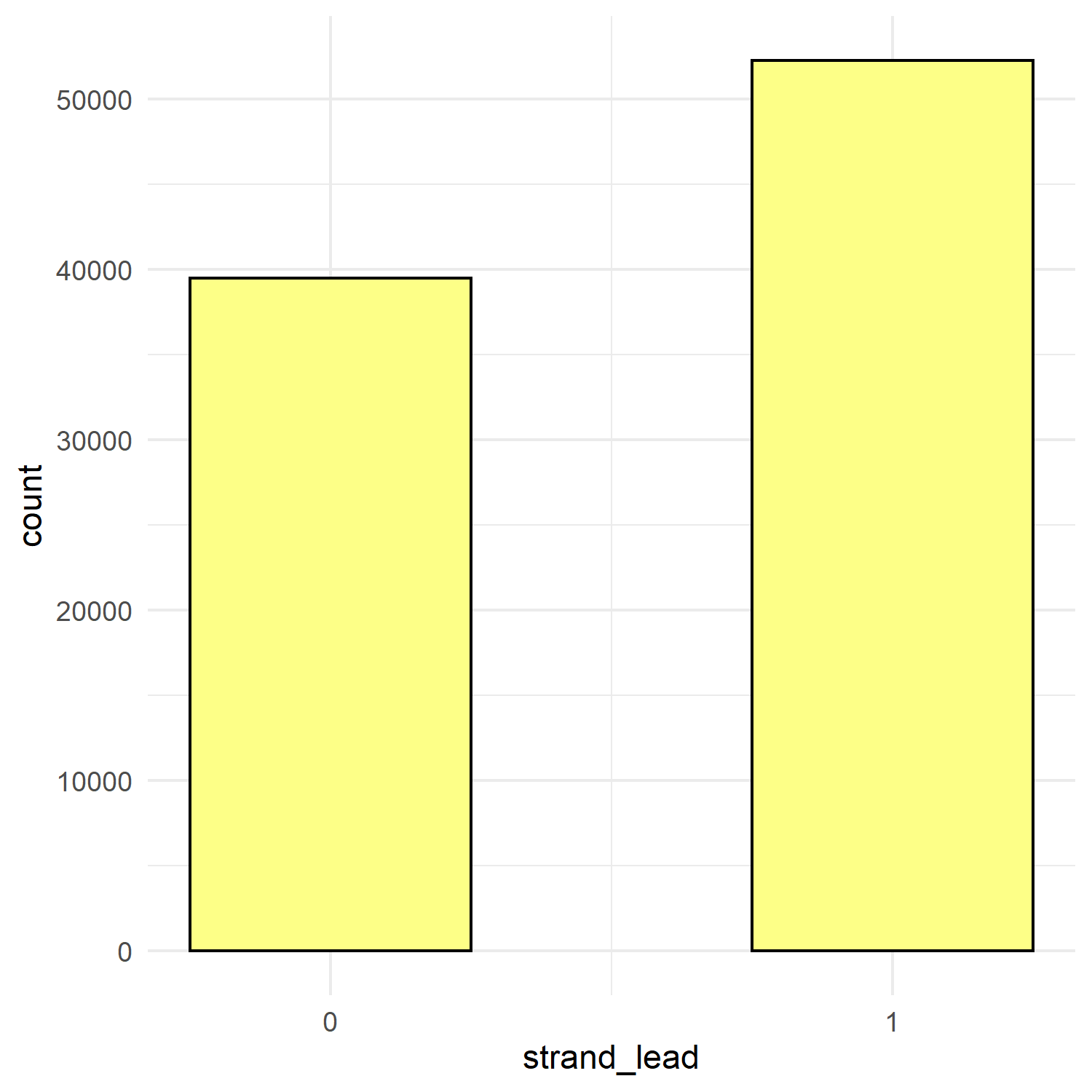 | 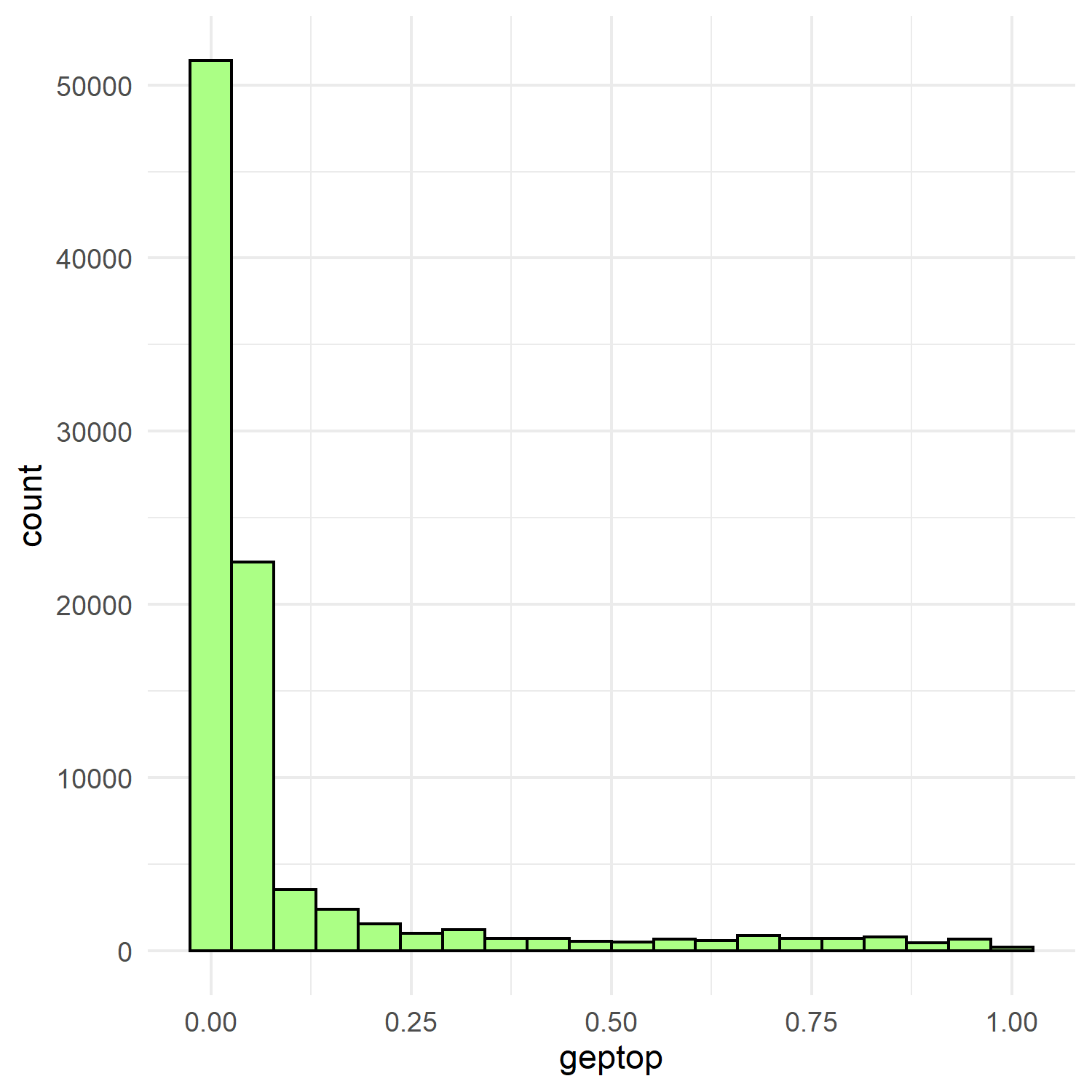 |
| --- | --- |
| 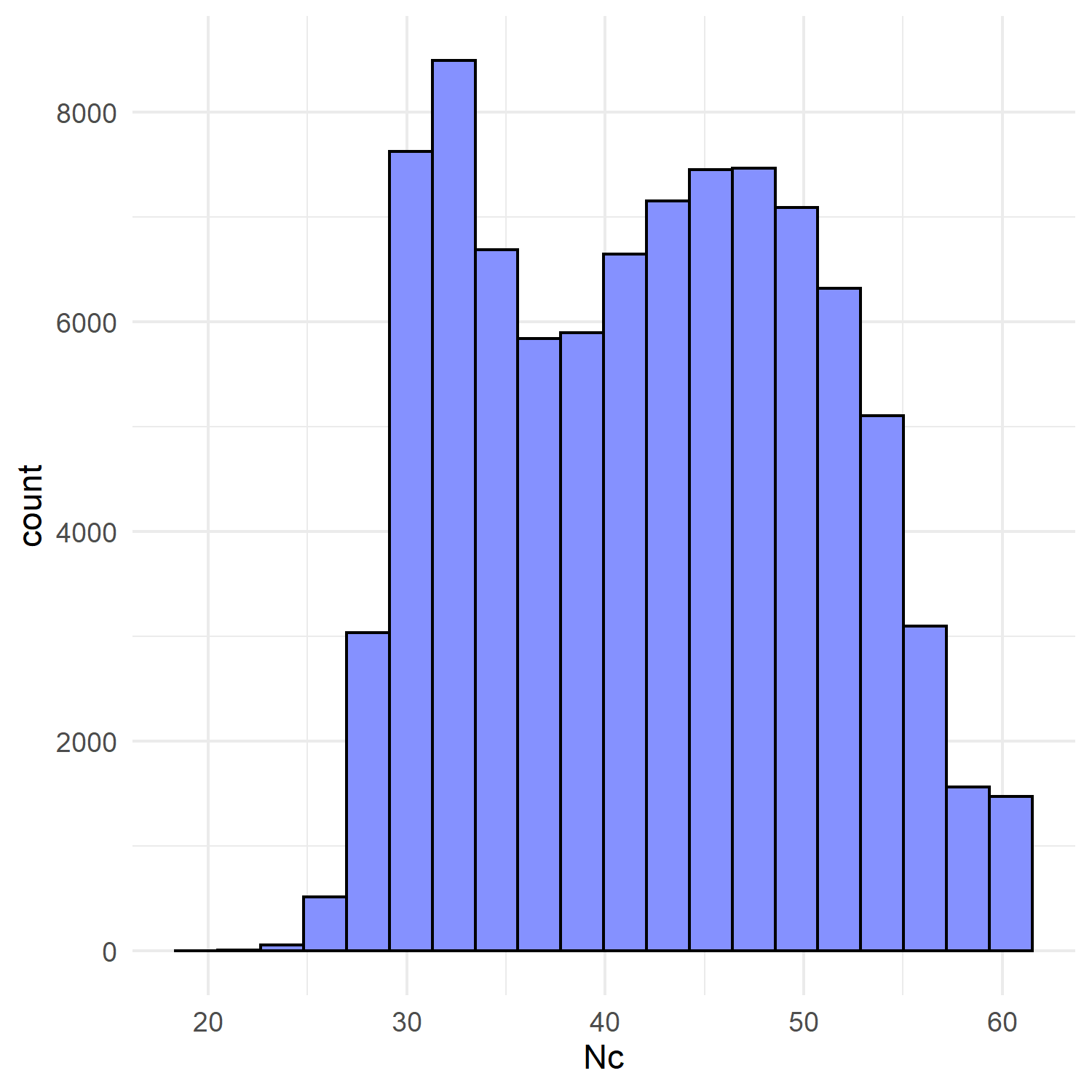 | 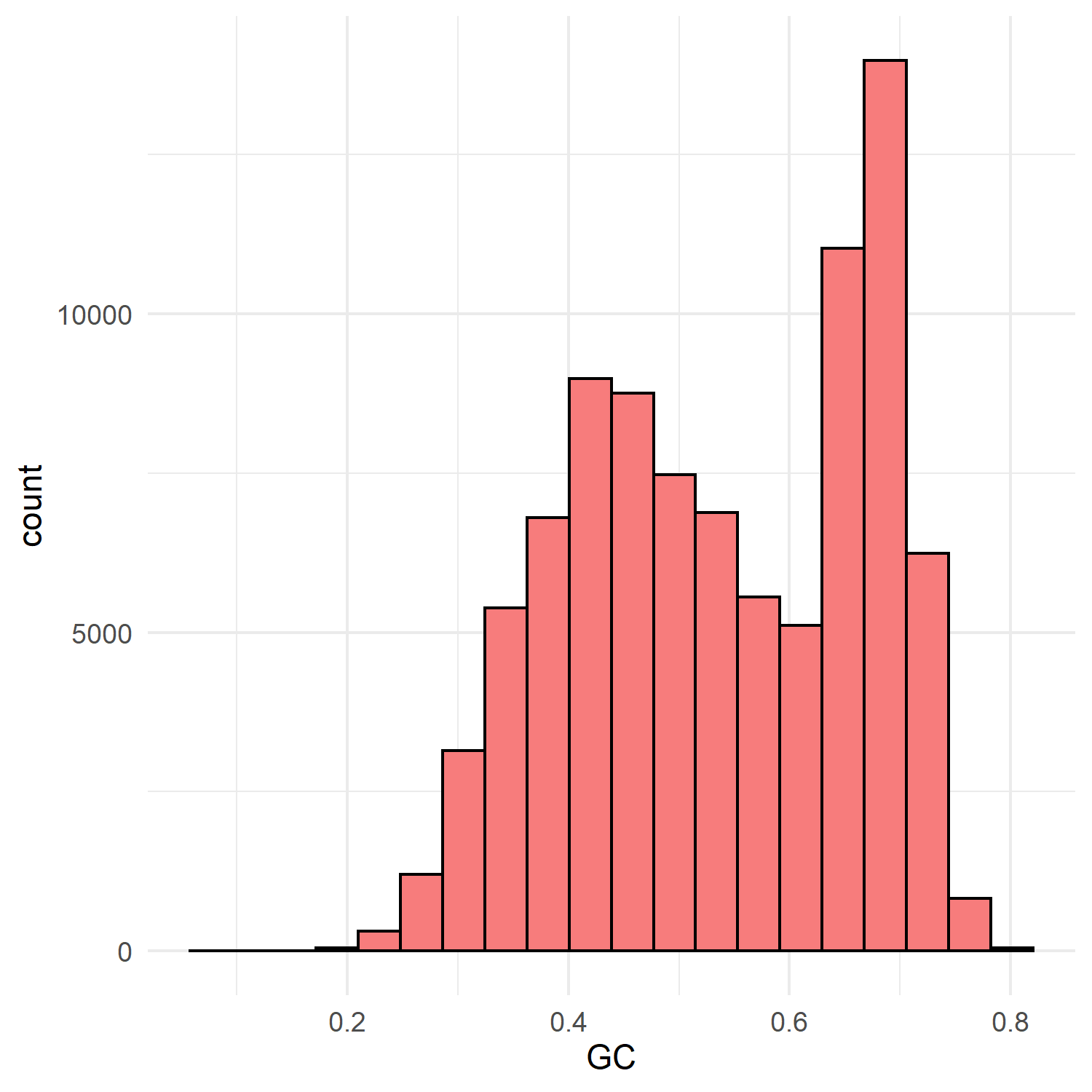 |
| 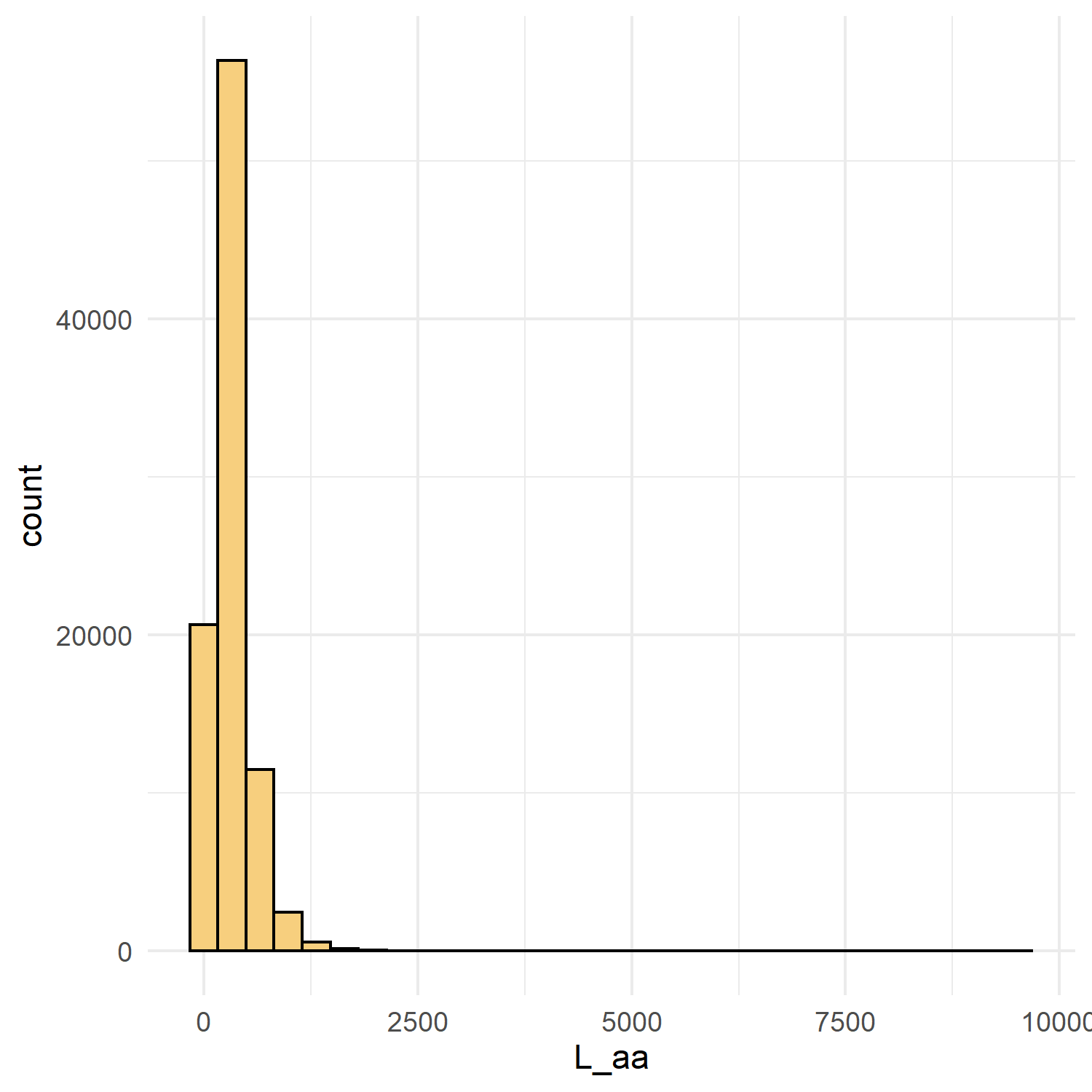 | 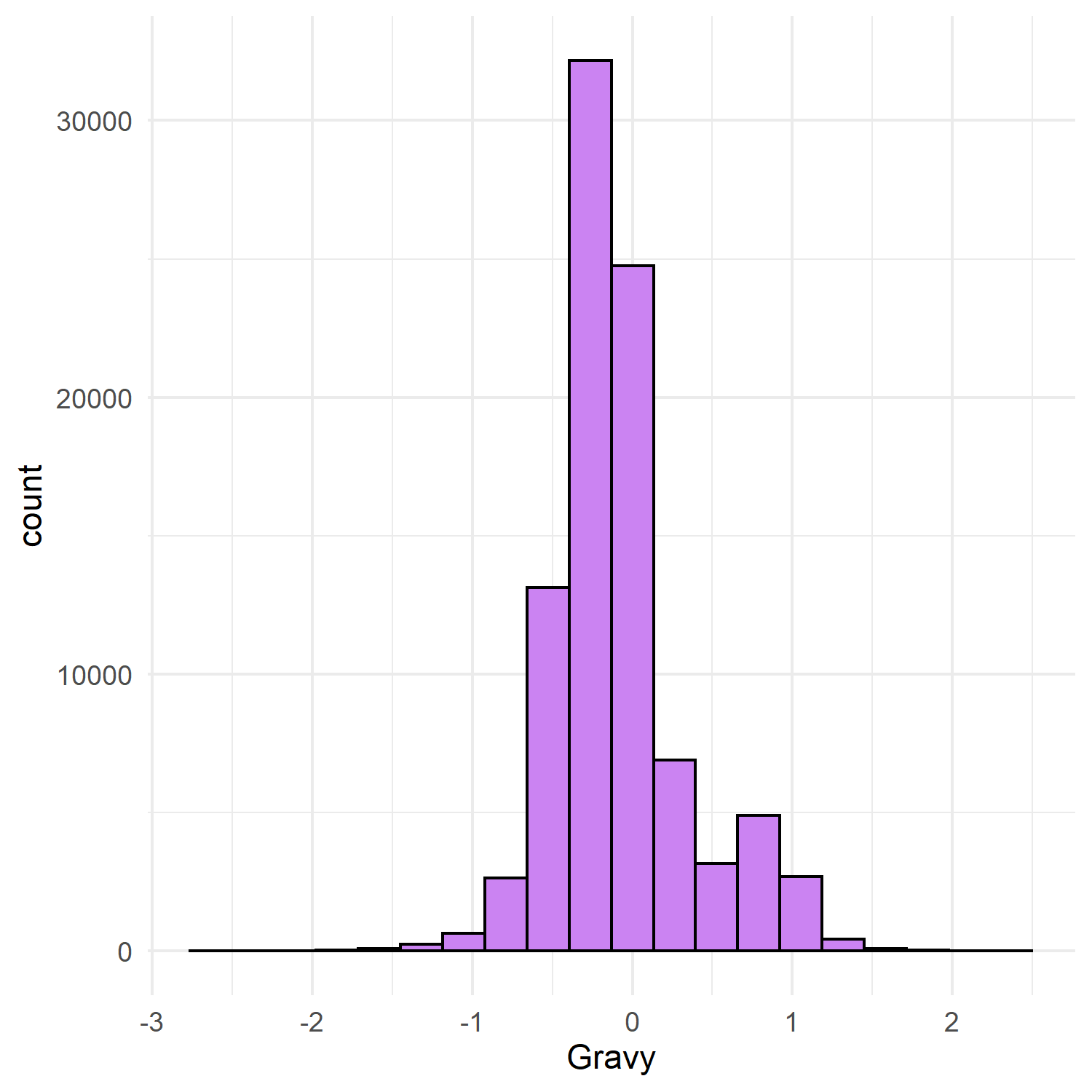 |

**Supplementary Figure S2**. Value distributions of the six computed features for all genes in the training and test sets.

**
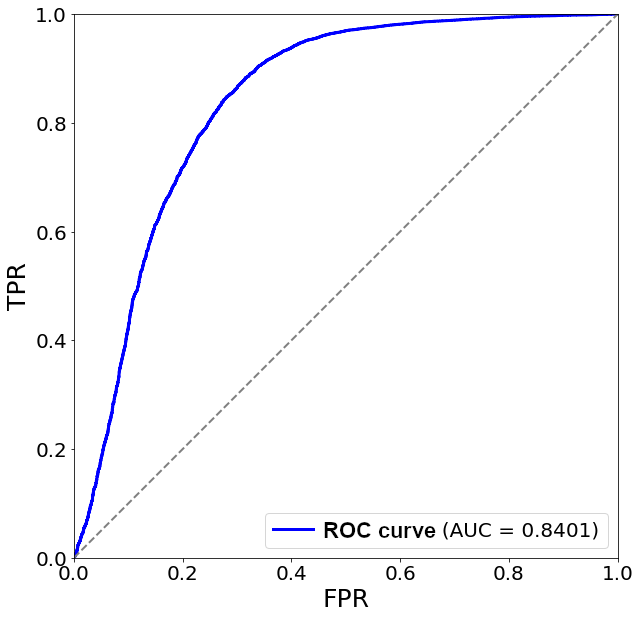
**

**Supplementary Figure S3**. ROC curve obtained by model evaluation on the test set, varying the input parameter *E* (essentiality score threshold).

**
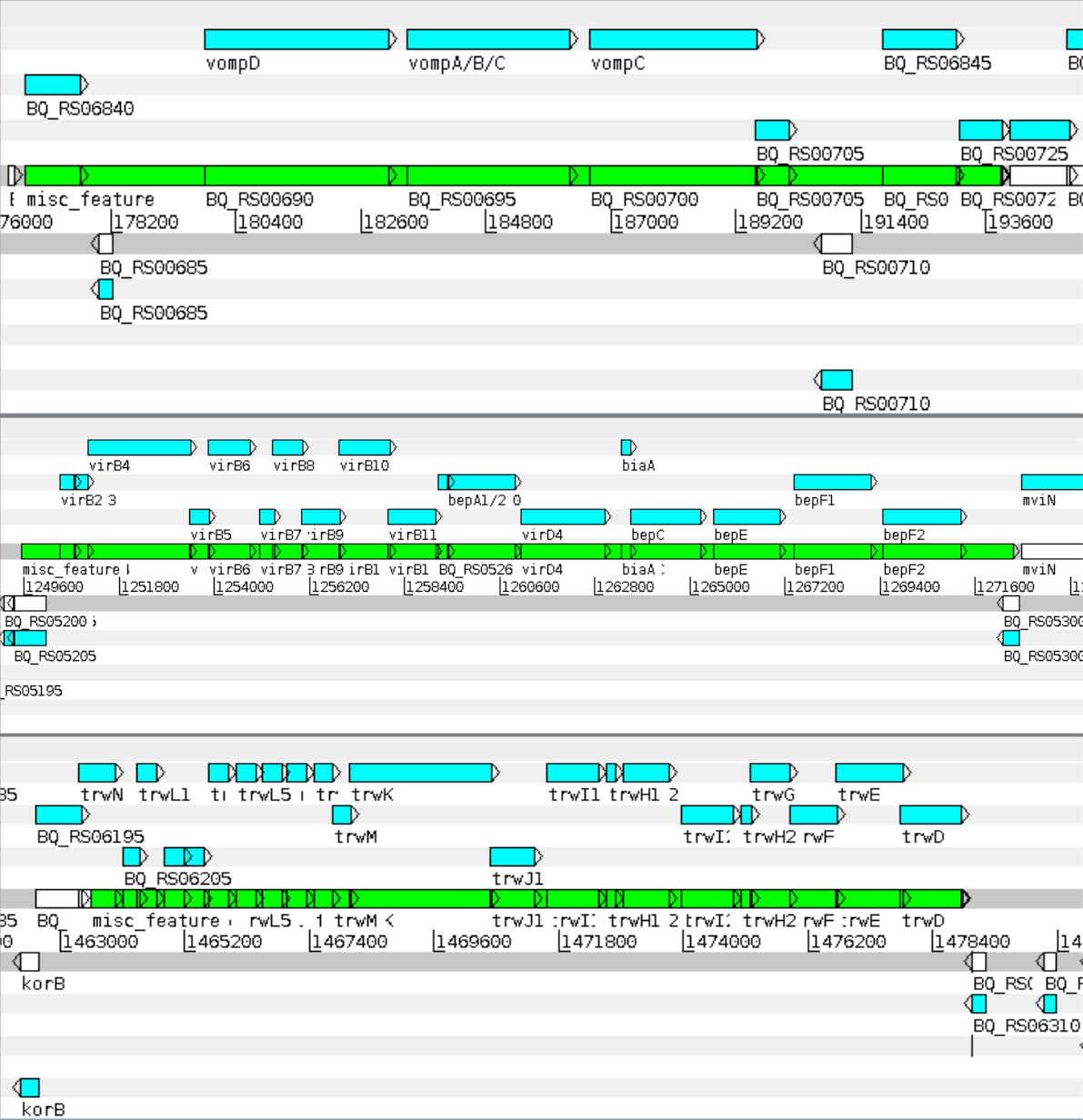
**

**Supplementary Figure S4**. Designed deletions (in green) which match pathogenicity islands in *B. quintana*’s genome, visualised in Artemis. D6 (176687 – 193839) matches the Vomp locus, D27 (1249540 – 1272464) the VirB/VirD4 locus, and D37 (1463545 – 1478903) the Trw locus.

**SUPPLEMENTARY REFERENCES**

Ara,K., Ozaki,K., Nakamura,K., Yamane,K., Sekiguchi,J. and Ogasawara,N. (2007) *Bacillus* minimum genome factory: effective utilization of microbial genome information. Biotechnol. Appl. Bioc., 46, 169–178.

Azhagesan,K., Ravindran,B. and Raman,K. (2018) Network-based features enable prediction of essential genes across diverse organisms. PLoS ONE, 13, e0208722.

Baumgart,M., Unthan,S., Kloß,R., Radek,A., Polen,T., Tenhaef,N., Müller,M.F., Küberl,A., Siebert,D., Brühl,N., et al. (2018) *Corynebacterium glutamicum* chassis C1*: building and testing a novel platform host for synthetic biology and industrial biotechnology. ACS Synth. Biol., 7, 132–144.

Baumgart,M., Unthan,S., Rückert,C., Sivalingam,J., Grünberger,A., Kalinowski,J., Bott,M., Noack,S. and Frunzke,J. (2013) Construction of a prophage-free variant of *Corynebacterium glutamicum* ATCC 13032 for use as a platform strain for basic research and industrial biotechnology. Appl. Environ. Microbiol., 79, 6006–6015.

Cheng,J., Xu,Z., Wu,W., Zhao,L., Li,X., Liu,Y. and Tao,S. (2014) Training set selection for the prediction of essential genes. PLoS One, 9, e86805.

da Silva, J.P.M., Acencio,M.L., Mombach,J.C.M., Vieira,R., da Silva,J.C., Lemke,N. and Sinigaglia,M. (2008) In silico network topology-based prediction of gene essentiality. Physica A, 387, 1049–1055.

Deng,J., Deng,L., Su,S., Zhang,M., Lin,X., Wei,L., Minai,A.A., Hassett,D.J. and Lu,L.J. (2011) Investigating the predictability of essential genes across distantly related organisms using an integrative approach. Nucleic Acids Res., 39, 795–807.

Fan,X., Zhang,Y., Zhao,F., Liu,Y., Zhao,Y., Wang,S., Liu,R. and Yang,C. (2020) Genome reduction enhances production of polyhydroxyalkanoate and alginate oligosaccharide in *Pseudomonas mendocina*. Int. J. Biol. Macromol., 163, 2023–2031.

Gustafson,A.M., Snitkin,E.S., Parker,S.C., DeLisi,C. and Kasif,S. (2006) Towards the identification of essential genes using targeted genome sequencing and comparative analysis. BMC Genomics, 7, 265.

Hashimoto,M., Ichimura,T., Mizoguchi,H., Tanaka,K., Fujimitsu,K., Keyamura,K., Ote,T., Yamakawa,T., Yamazaki,Y., Mori,H., et al. (2005) Cell size and nucleoid organization of engineered *Escherichia coli* cells with a reduced genome. Mol. Microbiol., 55, 137–149.

Hirokawa,Y., Kawano,H., Tanaka-Masuda,K., Nakamura,N., Nakagawa,A., Ito,M., Mori,H., Oshima,T. and Ogasawara,N. (2013) Genetic manipulations restored the growth fitness of reduced-genome *Escherichia coli*. J. Biosci. Bioeng., 116, 52–58.

Holman,A.G., Davis,P.J., Foster,J.M., Carlow,C.K. and Kumar,S. (2009) Computational prediction of essential genes in an unculturable endosymbiotic bacterium, *Wolbachia* of *Brugia malayi*. BMC Microbiol., 9, 243.

Hwang,Y.-C., Lin,C.-C., Chang,J.-Y., Mori,H., Juan,H.-F. and Huang,H.-C. (2009) Predicting essential genes based on network and sequence analysis. Mol. BioSyst., 5, 1672–1678.

Iwadate,Y., Honda,H., Sato,H., Hashimoto,M. and Kato,J. (2011) Oxidative stress sensitivity of engineered *Escherichia coli* cells with a reduced genome. FEMS Microbiol. Lett., 322, 25–33.

Kolisnychenko,V., Plunkett,G., Herring,C.D., Fehér,T., Pósfai,J., Blattner,F.R. and Pósfai,G. (2002) Engineering a reduced *Escherichia coli* genome. Genome Res., 12, 640–647.

Komatsu,M., Uchiyama,T., Ōmura,S., Cane,D.E. and Ikeda,H. (2010) Genome-minimized *Streptomyces* host for the heterologous expression of secondary metabolism. P. Natl. Acad. Sci. USA, 107, 2646–2651.

Li,Y., Lv,Y., Li,X., Xiao,W. and Li,C. (2017) Sequence comparison and essential gene identification with new inter-nucleotide distance sequences. J. Theor. Biol., 418, 84–93.

Li,Y., Zhu,X., Zhang,X., Fu,J., Wang,Z., Chen,T. and Zhao,X. (2016) Characterization of genome-reduced *Bacillus subtilis* strains and their application for the production of guanosine and thymidine. Microb. Cell Fact., 15, 94.

Lin,Y. and Zhang,R.R. (2011) Putative essential and core-essential genes in *Mycoplasma* genomes. Sci. Rep.-UK, 1, 53.

Lin,Y., Zhang,F., Xue,K., Gao,Y. and Guo,F. (2017) Identifying bacterial essential genes based on a feature-integrated method. IEEE ACM T. Comput. Bi., 16, 1274–1279.

Liu,X., He,T., Guo,Z., Ren,M. and Luo,Y. (2020) Predicting essential genes of 41 prokaryotes by a semi-supervised method. Anal. Biochem., 609, 113919.

Liu,X., Wang,B.-J., Xu,L., Tang,H.-L. and Xu,G.-Q. (2017) Selection of key sequence-based features for prediction of essential genes in 31 diverse bacterial species. PLoS One, 12.

Martínez-García,E., Nikel,P.I., Aparicio,T. and de Lorenzo,V. (2014) *Pseudomonas* 2.0: genetic upgrading of *P. putida* KT2440 as an enhanced host for heterologous gene expression. Microb. Cell Fact., 13, 159.

Mizoguchi,H., Sawano,Y., Kato,J. and Mori,H. (2008) Superpositioning of deletions promotes growth of *Escherichia coli* with a reduced genome. DNA Res., 15, 277–284.

Morimoto,T., Kadoya,R., Endo,K., Tohata,M., Sawada,K., Liu,S., Ozawa,T., Kodama,T., Kakeshita,H., Kageyama,Y., et al. (2008) Enhanced recombinant protein productivity by genome reduction in *Bacillus subtilis*. DNA Res., 15, 73–81.

Nigatu,D., Sobetzko,P., Yousef,M. and Henkel,W. (2017) Sequence-based information-theoretic features for gene essentiality prediction. BMC Bioinformatics, 18, 473.

Ning,L.W., Lin,H., Ding,H., Huang,J., Rao,N. and Guo,F.B. (2014) Predicting bacterial essential genes using only sequence composition information. Genet. Mol. Res., 13, 4564–4572.

Park,M.K., Lee,S.H., Yang,K.S., Jung,S.-C., Lee,J.H. and Kim,S.C. (2014) Enhancing recombinant protein production with an *Escherichia coli* host strain lacking insertion sequences. Appl. Microbiol. Biotechnol., 98, 6701–6713.

Plaimas,K., Eils,R. and König,R. (2010) Identifying essential genes in bacterial metabolic networks with machine learning methods. BMC Syst. Biol., 4, 56.

Pósfai,G., Plunkett,G., Fehér,T., Frisch,D., Keil,G.M., Umenhoffer,K., Kolisnychenko,V., Stahl,B., Sharma,S.S., Arruda,M. de, et al. (2006) Emergent properties of reduced-genome *Escherichia coli*. Science, 312, 1044–1046.

Reuß,D.R., Altenbuchner,J., Mäder,U., Rath,H., Ischebeck,T., Sappa,P.K., Thürmer,A., Guérin,C., Nicolas,P., Steil,L., et al. (2017) Large-scale reduction of the *Bacillus subtilis* genome: consequences for the transcriptional network, resource allocation, and metabolism. Genome Res., 27, 289–299.

Song,K., Tong,T. and Wu,F. (2014) Predicting essential genes in prokaryotic genomes using a linear method: ZUPLS. Integr. Biol. (Camb), 6, 460–469.

Suzuki,N., Nonaka,H., Tsuge,Y., Inui,M. and Yukawa,H. (2005) New multiple-deletion method for the *Corynebacterium glutamicum* genome, using a mutant lox sequence. Appl. Environ. Microbiol., 71, 8472–8480.

Suzuki,N., Nonaka,H., Tsuge,Y., Okayama,S., Inui,M. and Yukawa,H. (2005) Multiple large segment deletion method for *Corynebacterium glutamicum*. Appl Microbiol Biotechnol, 69, 151–161.

Wei,W., Ning,L.-W., Ye,Y.-N. and Guo,F.-B. (2013) Geptop: A gene essentiality prediction tool for sequenced bacterial genomes based on orthology and phylogeny. PLoS ONE, 8, e72343.

Westers,H., Dorenbos,R., van Dijl,J.M., Kabel,J., Flanagan,T., Devine,K.M., Jude,F., Séror,S.J., Beekman,A.C., Darmon,E., et al. (2003) Genome engineering reveals large dispensable regions in *Bacillus subtilis*. Mol. Biol. Evol., 20, 2076–2090.

Zhang,F., Huo,K., Song,X., Quan,Y., Wang,S., Zhang,Z., Gao,W. and Yang,C. (2020) Engineering of a genome-reduced strain *Bacillus amyloliquefaciens* for enhancing surfactin production. Microb. Cell Fact., 19, 223.

Zhu,D., Fu,Y., Liu,F., Xu,H., Saris,P.E.J. and Qiao,M. (2017) Enhanced heterologous protein productivity by genome reduction in *Lactococcus lactis* NZ9000. Microb. Cell Fact., 16, 1.

Zwiener,T., Dziuba,M., Mickoleit,F., Rückert,C., Busche,T., Kalinowski,J., Uebe,R. and Schüler,D. (2021) Towards a ‘chassis’ for bacterial magnetosome biosynthesis: genome streamlining of *Magnetospirillum gryphiswaldense* by multiple deletions. Microb. Cell Fact., 20, 35.
